# GRHL2-miR-200-ZEB1 maintains the epithelial status of ovarian cancer through transcriptional regulation and histone modification

**DOI:** 10.1038/srep19943

**Published:** 2016-02-18

**Authors:** Vin Yee Chung, Tuan Zea Tan, Ming Tan, Meng Kang Wong, Kuee Theng Kuay, Zhe Yang, Jieru Ye, Julius Muller, Cheryl M. Koh, Ernesto Guccione, Jean Paul Thiery, Ruby Yun-Ju Huang

**Affiliations:** 1 Cancer Science Institute of Singapore, National University of Singapore, Centre for Translational Medicine, 14 Medical Drive, #12-01, Singapore 117599; 2 Institute of Molecular and Cell Biology, A*STAR, 61 Biopolis Drive, Proteos, Singapore 13867; 3 Department of Biochemistry, Yong Loo Lin School of Medicine, National University of Singapore, MD 7, 8 Medical Drive, Singapore, 117596; 4 Department of Obstetrics and Gynaecology, National University Hospital, 5 Lower Kent Ridge Road, Singapore 119074; 5 Department of Anatomy, Yong Loo Lin School of Medicine, National University of Singapore, 14 Medical Drive, MD6, #14-02T, Singapore 117599

## Abstract

Epithelial-mesenchymal transition (EMT), a biological process by which polarized epithelial cells convert into a mesenchymal phenotype, has been implicated to contribute to the molecular heterogeneity of epithelial ovarian cancer (EOC). Here we report that a transcription factor—Grainyhead-like 2 (GRHL2) maintains the epithelial phenotype. EOC tumours with lower GRHL2 levels are associated with the Mes/Mesenchymal molecular subtype and a poorer overall survival. shRNA-mediated knockdown of GRHL2 in EOC cells with an epithelial phenotype results in EMT changes, with increased cell migration, invasion and motility. By ChIP-sequencing and gene expression microarray, microRNA-200b/a is identified as the direct transcriptional target of GRHL2 and regulates the epithelial status of EOC through ZEB1 and E-cadherin. Our study demonstrates that loss of GRHL2 increases the levels of histone mark H3K27me3 on promoters and GRHL2-binding sites at miR-200b/a and E-cadherin genes. These findings support GRHL2 as a pivotal gatekeeper of EMT in EOC via miR-200-ZEB1.

Besides being an important developmental program in morphogenesis, epithelial-mesenchymal transition (EMT) provides a mechanistic explanation for the progression of carcinoma in gaining metastatic properties^1^. In particular, the dissemination of epithelial ovarian cancer (EOC) has been suggested to involve repeated rounds of EMT and MET (the reverse of EMT) which render plasticity to the cancer cells^2^. This is supported by findings that associate EMT pathways with enhanced invasiveness, cancer stemness and chemoresistance in EOC^3^. These pathways trigger EMT through the activation of several key EMT transcription factors including SNAI1/2^4^, TWIST1/2^4^,^5^ and ZEB1/2^6^,^7^, which are mostly repressors of the epithelial marker E-cadherin^8^.

In our previous study^9^, Grainyhead-like 2 (GRHL2) emerged as a potential EMT transcription factor (TF) associated with the epithelial phenotype of EOC. GRHL2 is one of the three mammalian orthologs of the gene *grainyhead* identified in *Drosophila*^10^. Studies in mice have shown that all three mammalian Grainyhead-like TFs, Grhl1, Grhl2 and Grhl3, are required for normal embryonic development, of which Grhl2 and Grhl3 have important roles in neural tube closure^11^,^12^,^13^. Grhl2 transcriptionally modulates the expression of *Cdh1* (E-cadherin), *Cldn4* (Claudin 4), *Rab25* and *Nkx2-1*, which are crucial for the differentiation and junctional maintenance of epithelial cells^12^,^14^,^15^. In zebrafish, the role of Grhl2b in regulating *claudin b* and *epcam* determines proper otic development and hearing function^16^. Some of these Grhl2 target genes have been validated in a study in human lung epithelium^17^.

In recent years, GRHL2 has been implicated in cancer progression. GRHL2 is overexpressed in oral squamous cell carcinoma (OSCC) and it confers a growth advantage by positively regulating telomerase^18^. In breast cancer, GRHL2 acts as an EMT suppressor^19^ by forming a double negative feedback loop with the EMT driver ZEB1^20^,^21^,^22^, and is involved in tumourigenesis^21^,^22^,^23^. The role of GRHL2 in regulating tumour growth has also been demonstrated in gastric cancer^24^ and colorectal cancer^25^. Studies suggest that the aberration of GRHL2 expression in cancer arises from genomic alterations, as *GRHL2* resides in 8q22.3 region, which is frequently amplified in hepatocellular cancer (HCC), breast cancer, lung cancer, ovarian cancer and melanoma^26^,^27^. Within this 8q22.3 gene cluster, *GRHL2* and *UBR5* have been shown, through their respective proteins, to suppress death receptor-induced apoptosis in cancer cells^27^. Besides the finding of 8q22.3 amplification in ovarian cancer^27^, data from TCGA (The Cancer Genome Atlas Research Network) also showed *GRHL2* amplification in about 8% to 22% of ovarian serous cystadenocarcinoma^28^,^29^. To date, the functional roles of GRHL2 in EOC have yet to be elucidated.

## Results

### GRHL2 expression in EOC cell lines and tumours correlates with the Epithelial phenotype and is associated with better patient survival

**Figure 1:**
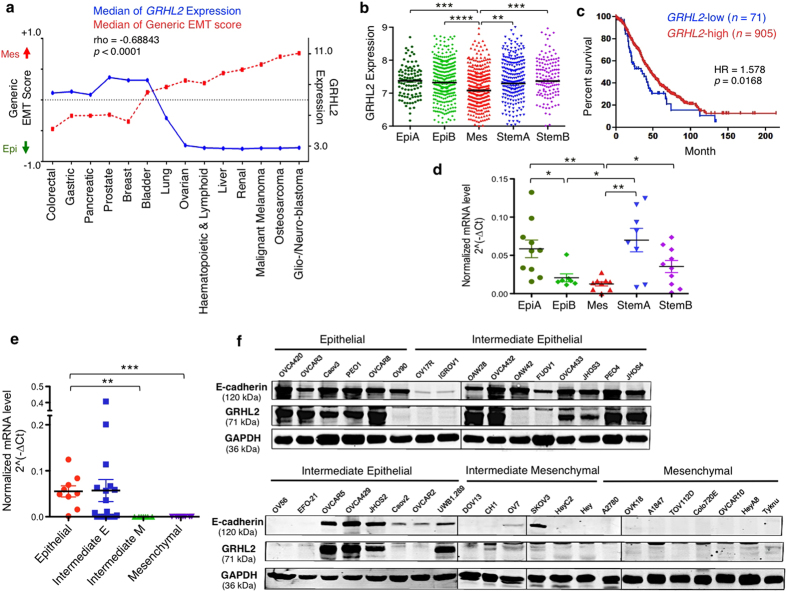
Correlation of GRHL2 expression with EMT score, molecular subtype and EMT phenotype. (**a**) The median generic EMT score (blue) and the median *GRHL2* expression (red) of different cancer types from the Cancer Cell Line Encyclopedia (CCLE). EMT score nearer to +1.0 is more mesenchymal-like (Mes), whereas an EMT score nearer to −1.0 is more epithelial-like (Epi). Correlation between the two variables was checked by Spearman’s correlation test (*n* = 807). (**b**) The mRNA expression of *GRHL2* (mean ± SEM) in EOC tumours of five molecular subtypes: EpiA, EpiB, Mes, StemA and StemB. The Mes subgroup showed significant lower expression, based on unpaired *t-*tests. **p < 0.01; ***p < 0.001; ****p < 0.0001. (**c**) Kaplan-Meier curves showing overall survival (% survival) in patients with *GRHL2*-high (red) and *GRHL2*-low (blue) tumours using a cutoff gene expression level matched with the protein expression in cell lines. (**d**) The normalized mRNA expression of *GRHL2* (2^−∆Ct^), as measured by RT-qPCR in 44 archived EOC samples classified into the five molecular subtypes (mean ± SEM). Unpaired *t-*tests were performed. *p < 0.05; **p < 0.01. (**e**) The normalized mRNA expression of *GRHL2* (2^−∆Ct^) (duplicates) measured by RT-qPCR in the SGOCL collection of EOC cell lines (*n* = 42) classified into four phenotypes: Epithelial, Intermediate E, Intermediate M and Mesenchymal. Unpaired *t-*tests were performed. **p < 0.01; ***p < 0.001. (**f**) Western blots showing the protein expression of GRHL2, E-cadherin and GAPDH in 38 EOC cell lines representing the four phenotypes. Full-length blots are presented in Supplementary Fig. S10.

Based on the EMT scoring scheme in our previous study^30^ and the transcriptomic data of Cancer Cell Line Encyclopedia (CCLE)^31^, we found that cancer types with lower EMT scores (more epithelial-like) had higher *GRHL2* expression whereas cancer types with strong mesenchymal features had lower *GRHL2* expression (Fig. 1a). Overall, the expression of *GRHL2* correlated negatively with the generic EMT score in CCLE cell lines. However, within a cancer type such as EOC, the expression of *GRHL2* was heterogeneous. Therefore, we analyzed *GRHL2* expression within EOC tumours across the five molecular subtypes—Epithelial-A (EpiA), Epithelial-B (EpiB), Mesenchmal (Mes), Stem-like-A (StemA), Stem-like-B (StemB)^32^. A significantly lower expression of *GRHL2* was observed in the Mes subtype (Fig. 1b). In addition, *GRHL2*-high tumours showed better overall survival (OS) in patients as compared with *GRHL2*-low tumours (Hazard Ratio = 1.578; *p* = 0.0168) (Fig. 1c). In an independent EOC collection (JPKO, GSE30311), the median mRNA level of *GRHL2* in Mes tumours was also significantly lower, as validated by RT-qPCR (Fig. 1d). The mRNA expression of *GRHL2* was then analyzed in a panel of EOC cell lines (SGOCL) that were classified into four phenotypes forming an EMT Spectrum: Epithelial (E), Intermediate E (IE), Intermediate M (IM) and Mesenchymal (M)^9^. The mRNA level of *GRHL2* correlated negatively with the EMT Spectrum, showing significantly higher expression in epithelial-like phenotypes (E and IE) and low to undetectable levels in mesenchymal-like phenotypes (IM and M) (Fig. 1e). Western blotting of 38 representative cell lines showed that the protein level of GRHL2 correlated with that of E-cadherin, with low or undetectable GRHL2 in the IM and M lines (Fig. 1f). These results suggest that GRHL2 is associated with the epithelial-like phenotype of EOC.

### GRHL2 knockdown induces sequential EMT changes along the EMT spectrum

**Figure 2:**
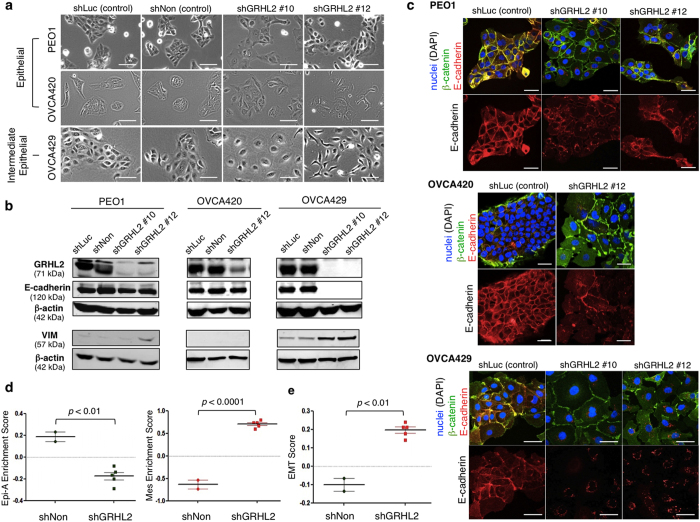
GRHL2 is required for the epithelial phenotype of EOC and the distribution of E-cadherin at cell–cell junctions. (**a**) The morphologies of EOC cell lines (PEO1, OVCA420, OVCA429) stably infected with control shRNAs (shLuc, shNon) and two different GRHL2-targeting shRNAs (shGRHL2 #10, shGRHL2 #12). Scale = 100 μm. (**b**) Western blots of GRHL2, E-cadherin, vimentin and β-actin in EOC stable cell lines. Upper bands in the GRHL2 panel were deemed non-specific. Full-length blots are presented in Supplementary Fig. S10. (**c**) Immunofluorescence stainings of E-cadherin (red) and β-catenin (green) in the EOC stable cell lines. Nuclei were stained blue (DAPI). Scale = 50 μm. (**d**) Scatter plots showing molecular subtype enrichment scores for OVCA429 shNon (*n* = 2) and shGRHL2 cells (shGRHL2 #10, *n* = 2 and shGRHL2 #12, *n* = 3) using single-sample Gene Set Enrichment Analysis (ssGSEA), based on microarray gene expression. Bars indicate mean values ± SEM. Unpaired *t*-tests were used. (**e**) EMT enrichment scores of OVCA429 shGRHL2 cells were higher as compared with that of shNon cells, based on unpaired *t-*tests. Bars indicate mean values ± SEM.

GRHL2 was stably knocked-down using two GRHL2-targeting shRNAs (shGRHL2 #10 and shGRHL2 #12) in three cell lines: two belong to the E phenotype (PEO1, OVCA420) and one to the IE phenotype (OVCA429). shLuciferase (shLuc) and non-targeting shRNA (shNon) were used as controls. The control PEO1 and OVCA420 cells formed tight epithelial colonies whereas the control OVCA429 cells displayed a more spread-out morphology, reflecting its intermediate phenotype (Fig. 2a). shGRHL2 PEO1 and OVCA420 cells formed less-compact colonies, whereas shGRHL2 OVCA429 cells became more scattered, with a flattened (shGRHL2 #10) or a more spindle-shaped morphology (shGRHL2 #12). OVCA420 shGRHL2 #10 cells did not survive after subsequent passaging; thus, experiments hereafter for OVCA420 involved only shGRHL2 #12.

After GRHL2 knockdown, a reduction of E-cadherin protein was seen in the IE line, OVCA429, but not in the E lines, PEO1 and OVCA420 (Fig. 2b). Intriguingly, immunofluorescence (IF) stainings of PEO1 and OVCA420 cells showed weaker E-cadherin signals at cell–cell junctions in shGRHL2 cells, accompanied by a fraction of cytoplasmic E-cadherin that was uncoupled with β-catenin (Fig. 2c). In OVCA429 cells, junctional E-cadherin was no longer observed in both shGRHL2 #10 and shGRHL2 #12, leaving some cytoplasmic E-cadherin around the nuclei (Fig. 2c) that was co-localized with the autophagosome marker LC3A (Supplementary Fig. S1). These results suggest that loss of GRHL2 might abrogate the subcellular distribution of E-cadherin in cells undergoing partial EMT. Consistently, GRHL2 knockdown significantly reduced the mRNA level of *CDH1* (E-cadherin) in OVCA429 but not in PEO1 and OVCA420 (Supplementary Fig. S2a). In OVCA420 cells, protein levels of vimentin were low or undetectable (not shown), whereas, in PEO1 and OVCA429 cells, GRHL2 knockdown resulted in stronger vimentin protein bands (Fig. 2b) and immunofluorescence signals (Supplementary Fig. S2b). In summary, GRHL2 knockdown in PEO1 may lead to a phenotype switch from E to IE through an altered E-cadherin subcellular distribution and up-regulation of vimentin. In OVCA429, GRHL2 knockdown may further switch the cells from IE to IM via the loss of junctional E-cadherin and the up-regulation of vimentin (Supplementary Fig. S2e). In another IE cell line OVCAR5, although knockdown of GRHL2 did not affect the total E-cadherin expression, we observed similar changes in cell morphology and disrupted membrane localization of E-cadherin at cell-cell junctions (Supplementary Fig. S3).

### Knockdown of GRHL2 alters EMT signatures and switches the molecular subtype of EOC

We performed gene expression microarray on OVCA429 shNon, shGRHL2 #10 and shGRHL2 #12 cells to assess transcriptomic changes. GRHL2-knockdown cells showed increased expression of genes usually down-regulated in the EpiA subtype, reduced expression of genes usually enriched in EpiA, and increased expression of signature genes that correlate with the Mes subtype (Supplementary Table S1). Using single-sample Gene Set Enrichment Analysis (ssGSEA)^33^ to determine the molecular subtype and EMT score^32^,^34^, we found that the molecular subtype of OVCA429 changed from EpiA (shNon) to Mes for both shGRHL2 #10 and shGRHL2 #12 cells (Fig. 2d). This was further confirmed by the EMT scores, classifying control cells (shNon) as Epithelial, with an average EMT score of −0.1013, shGRHL2 #10 and shGRHL2 #12 stable cells as Mesenchymal with a combined average score = 0.1968) (Fig. 2e). Hence, knockdown of GRHL2 affected the expression of EMT signatures and resulted in a molecular subtype switch in OVCA429 cells.

### Knockdown of GRHL2 promotes cell migration, invasion and secretion of matrix metalloproteinase 2 (MMP2) in EOC

**Figure 3:**
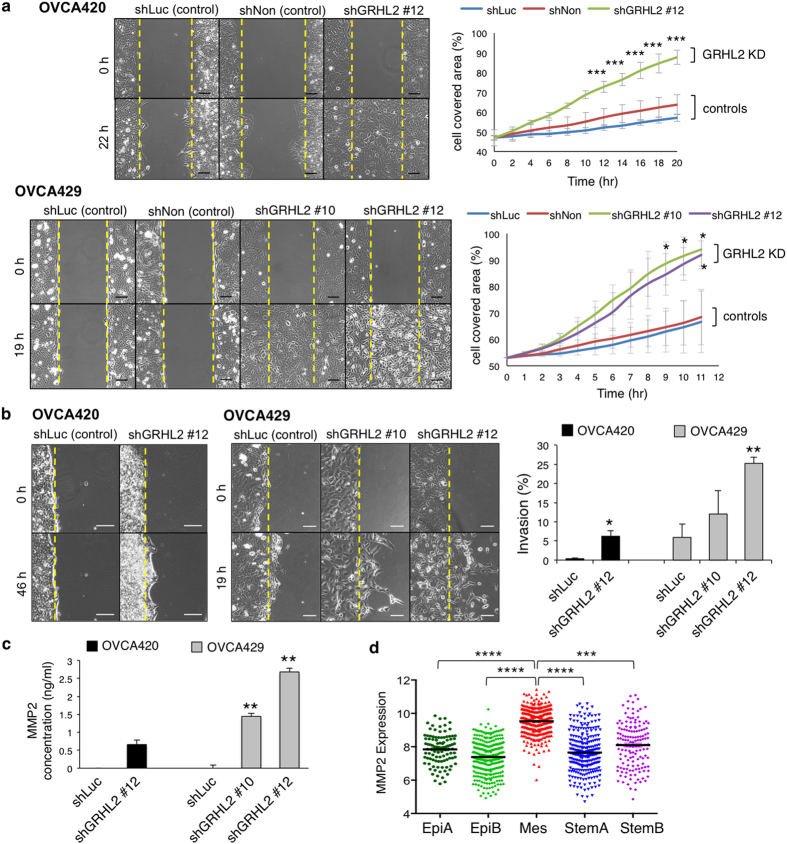
Knockdown of GRHL2 promotes cell migration, invasion and MMP2 secretion in EOC cells. (**a**) Gap closure assays (left) showing migration of controls (shLuc, shNon) and GRHL2-knockdown OVCA420 (shGRHL2 #12) and OVCA429 (shGRHL2 #10, #12) cells before (0 h) and after 22 h (OVCA420) or 19 h (OVCA429). Yellow lines mark initial edges of the gaps. Scale = 100 μm. Line graphs (right) showing the percentage of cell-covered area over time in the stable cell lines. Mean ± SEM from two (OVCA420) or three (OVCA429) independent experiments. Two-way ANOVA and Bonferroni *post-hoc* tests were used; *p* < 0.05; ***p < 0.001. (**b**) Cell invasion in Matrigel before (0 h) and after 46 h (OVCA420) or 19 h (OVCA429). Yellow lines mark the initial edges. Scale = 100 μm. Bar chart (right) showing percentage of area invaded in OVCA420 (black) and OVCA429 (grey) stable cell lines. Mean ± SEM from two independent experiments. Unpaired *t*-tests; *p < 0.05; **p < 0.01. (**c**) The MMP2 levels in conditioned media of control (shLuc) and GRHL2-knockdown cells of OVCA420 (black) and OVCA429 (grey), as measured by quantitative ELISA. Unpaired *t*-tests were used; **p < 0.01. Mean ± SEM from two independent experiments. (**d**) The *MMP2* expression (mean ± SEM) in EOC tumours of different molecular subtypes. Unpaired *t-*tests; ***p < 0.001, **** *p* < 0.0001.

From time-lapse imaging, PEO1 showed the slowest basal motility as compared with OVCA420 and OVCA429, and the knockdown of GRHL2 in all three cell lines led to an increased speed of individual cell locomotion (Supplementary Fig. S4a). In gap closure migration assays, the inherently slow-moving PEO1 cells did not show significant change in migration after GRHL2 knockdown (data not shown). On the other hand, GRHL2 knockdown in OVCA420 and OVCA429 cells resulted in an increased speed of gap closure (Fig. 3a). At the migrating front, detached cells were observed in shGRHL2 of OVCA420 and OVCA429; this is in contrast with that seen in the control cells, which remained attached to neighbouring cells and engaged in collective migration (Supplementary Fig. S4b). Overall, these findings suggest a change in the migratory behavior of shGRHL2 cells. We further examined the organization of F-actin and the phosphorylation of myosin II at serine residue 19 (pS19-MLC2). Compared with control cells, which showed pS19-MLC2 at the edge of cell colonies, the pS19-MLC2 signals in shGRHL2 cells were more concentrated at intercellular borders (Supplementary Fig. S4c). Similar changes were observed in OVCAR5 cells after GRHL2 knockdown (Supplementary Fig. S3b). Thus, GRHL2 could be involved in the regulation of actomyosin contractility. Suppression of actomyosin activity at cell–cell contacts has been reported to be a requirement for collective cell migration^35^. Therefore, the observed changes in GRHL2-knockdown cells in this study suggest a switch from collective to single-cell migration. This is in line with a study of murine lung epithelial cells which also reported a change from collective cell migration to detached cell migration in clusters after Grhl2 knockdown^15^.

In a modified Matrigel-embedded gap invasion assay, shGRHL2 OVCA420 and OVCA429 cells showed increased rate of invasion into the Matrigel matrix (Fig. 3b). The control cells displayed a collective invasive front whereas the shGRHL2 cells penetrated the matrix as individual cells. Interestingly, the increased cell invasiveness correlated with the levels of secreted matrix metalloproteinase-2 (MMP2) in shGRHL2 OVCA429 cells (Fig. 3c). With reference to the microarray meta-analysis of the EOC tumours, the endogenous expression of *MMP2* in the Mes subtype was found to be the highest (Fig. 3d), suggesting that MMP2 is a mesenchymal-associated MMP in EOC.

The observed changes in cell migration and invasion were not due to increased cell proliferation (Supplementary Fig. S5a). Although GRHL2 has been reported to increase anoikis sensitivity and suppress mammosphere formation in mammary epithelial cells^19^, we did not observe any significant change in anoikis sensitivity or spheroid growth in GRHL2-knockdown EOC cells (Supplementary Fig. S5b,c). This suggests that EMT induced by the loss of GRHL2 might not be sufficient to overcome anchorage dependency in EOC.

### Genome-wide identification of GRHL2 target genes in EOC cells

**Figure 4:**
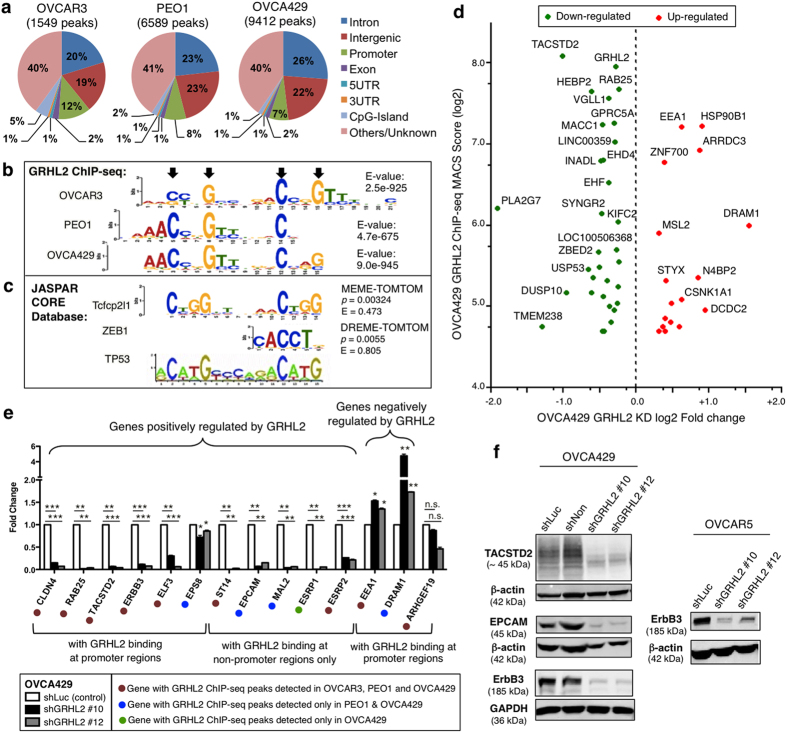
Genome-wide identification of GRHL2 targets in EOC. (**a**) GRHL2 ChIP-seq peaks in OVCAR3, PEO1 and OVCA429 cell lines classified based on human genomic annotations (hg19). (**b**) DNA-binding motifs of GRHL2 generated by MEME-ChIP analyses from our GRHL2 ChIP-seq data. Black arrows mark the invariant C and G bases. (**c**) DNA-binding motifs of transcription factors Tcfcp2l1 (murine), Zeb1 (chicken) and TP53 (human) from JASPAR database^56^. The *p*-values and E-values were obtained from Tomtom analyses comparing DNA-binding motif of GRHL2 with Tcfcp2l1 or ZEB1. (**d**) Cross-analysis of GRHL2 ChIP-seq and gene expression microarray of OVCA429 stable cell lines. Potential direct target genes of GRHL2 are represented as green dots (positively regulated by GRHL2) and red dots (negatively regulated by GRHL2) corresponding to ChIP-seq MACS score (*y*-axis) and gene expression fold change (*x*-axis), both in log2-transformed values. (**e**) The mRNA expression of *CLDN4, RAB25, TACSTD2, ERBB3, ELF3, EPS8, ST14, EPCAM, MAL2, ESRP1/2, EEA1, DRAM1* and *ARHGEF19* as analyzed by RT-qPCR in control (shLuc, white) and GRHL2-knockdown (shGRHL2 #10, black; shGRHL2 #12, grey) OVCA429 cells. The fold change (2^−∆∆Ct^) for each gene was calculated with respect to shLuc as a control. Unpaired *t*-tests were performed on the 2^−∆Ct^ values, n.s. = not significant; *p < 0.05; **p < 0.01; ***p < 0.001. (**f**) Western blots (left) of TACSTD2, EPCAM and ErbB3 in control (shLuc, shNon) and GRHL2-knockdown (shGRHL2 #10, #12) OVCA429 cells. Western blots (right) of ErbB3 in control (shLuc) and GRHL2-knockdown (shGRHL2 #10, #12) OVCAR5 cells. Full-length blots are presented in Supplementary Fig. S10.

To identify GRHL2 target genes, we performed chromatin immunoprecipitation-sequencing (ChIP-seq) in three EOC cell lines with high endogenous expression of GRHL2: OVCAR3 and PEO1 of the E phenotype, and OVCA429 of the IE phenotype. ChIP-seq peaks were called by MACS (Model-based analysis of ChIP-seq)^36^ and highly enriched peaks were shortlisted as GRHL2 binding sites. GRHL2 binding sites were found at gene promoters, introns, exons, intergenic regions and other/unknown regions (Fig. 4a). Overall, OVCA429 of the IE phenotype had the most number of GRHL2 binding sites associated with the most number of genes. Many target genes showed more than one GRHL2 binding site across the gene coding/non-coding regions, suggesting that the binding of GRHL2 to multiple DNA regions (promoters/enhancers) may involve long-distance chromatin interactions.

GRHL2 has been reported to share a similar DNA-binding motif (AACCGGTT) with other GRHL family members^10^,^17^,^37^ but also shows variability for some target genes^15^,^38^. Using MEME-ChIP analysis (Multiple Expectation Maximization for Motif Elicitation), we generated the DNA-binding motif of GRHL2 from ChIP-seq peaks with high MACS scores. The motifs resembled the consensus GRHL sequence, but with two core CNNG motifs occurring in tandem, spaced by five bases (Fig. 4b). This DNA-binding motif of GRHL2 showed similarities to the binding motif of Tfcp2l1 from the CP2 family (paralogous to GRHL2), and of ZEB1, which is a key EMT-activating transcription factor (Fig. 4c). The two CNNG motifs in tandem also resembled the binding motif of p53 family (two CNNG spaced by six bases), which was reported to be phylogenetically related to GRH/CP2 family based on protein structure and folding^39^.

GRHL2 has been shown to bind to the intronic enhancer of *CDH1* (E-cadherin) and the promoters of *CLDN4*, *RAB25, ARHGEF19* and *ERBB3* for transcriptional activation^12^,^14^,^17^,^21^,^37^. Our ChIP-seq data showed GRHL2 binding at the intron 2 of *CDH1* and the promoter regions of *CLDN4*, *RAB25, ERBB3* and *ARHGEF19* (Supplementary Table S2). The mesenchymal-related EMT driver *ZEB1* has also been reported to be directly repressed by GRHL2 in mammary epithelial cells^19^. However, no GRHL2 binding was detected at the promoter region of *ZEB1*. Instead, a ChIP-seq peak with a relatively lower enrichment/score was found within intron 1 of *ZEB1* in OVCA429 (Supplementary Table S2). By ChIP-qPCR, we validated that *CDH1* and *RAB25* are direct targets of GRHL2, whereas *ZEB1* showed no or low enrichment of GRHL2 binding at its promoter (Supplementary Fig. S6).

To identify other GRHL2 targets, we combined the gene expression microarray data with the GRHL2 ChIP-seq data of OVCA429 cells (Fig. 4d), followed by RT-qPCR validation. Epithelial-related genes, such as *CLDN4, RAB25*, *TACSTD2, ERBB3* and *ELF3*, which showed GRHL2 binding at their respective promoters, were markedly down-regulated after GRHL2 knockdown (Fig. 4e), with the exception of *EPS8*, which showed a relatively modest down-regulation. Other epithelial-related genes that were down-regulated after GRHL2 knockdown—*CDH1, ST14, EPCAM, MAL2, ESRP1* and *ESRP2* (Fig. 4e and Supplementary Fig. S2a)—have GRHL2 binding sites at intronic or distal intergenic regions (Supplementary Table S2). *EEA1* and *DRAM1*, on the other hand, could be genes repressed by GRHL2, evident by the increased expression seen in shGRHL2 cells (Fig. 4e) and the GRHL2 binding at their promoter regions (Supplementary Table S2). Western blots further confirmed that the protein levels of TACSTD2, EPCAM and ErbB3 were down-regulated in GRHL2-knockdown cells (Fig. 4f). The down-regulation of ErbB3 protein in GRHL2-knockdown cells (both in OVCA429 and OVCAR5) suggests GRHL2 as a regulator of the ErbB3 signaling pathway, which is consistent with reported findings in breast cancer and ovarian cancer cells^12^,^40^.

### GRHL2 regulates *MIR200B* and *MIR203A* in EOC cells

**Figure 5:**
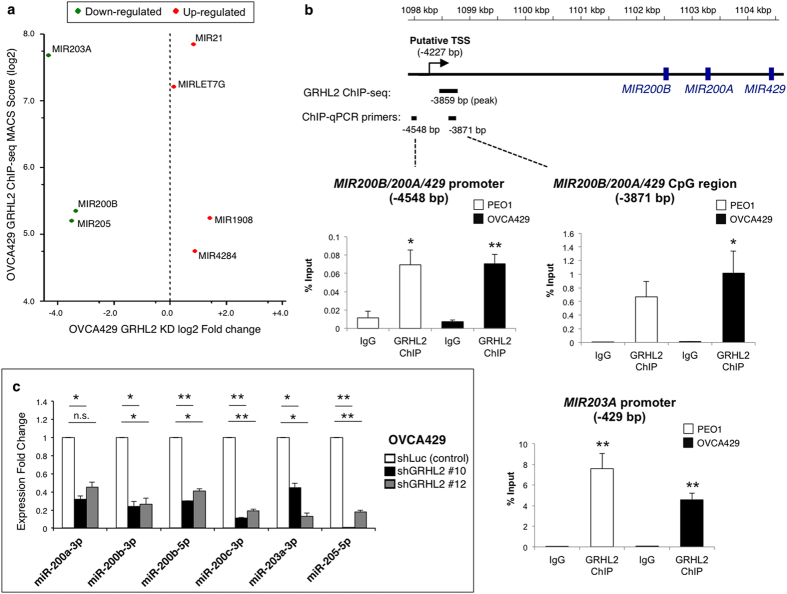
GRHL2 binds to and regulates *MIR203A* and *MIR200B*. (**a**) A cross-analysis of GRHL2 ChIP-seq and miRNA expression microarray of OVCA429 stable cell lines. Potential target miRNA genes regulated by GRHL2 are represented as green dots (positively regulated by GRHL2) and red dots (negatively regulated by GRHL2) corresponding to ChIP-seq MACS (model-based analysis of ChIP-seq) score (*y*-axis) and expression fold change (*x*-axis), both in log2 transformed values. (**b**) ChIP-qPCR of IgG (control) and GRHL2 at the promoter and CpG region upstream of the *MIR200B/200A/429* cluster and at the promoter of *MIR203A*. Signals of IgG control and ChIP samples were normalized to input DNA and presented as % input with SEM from biological triplicates. Unpaired *t*-tests with respect to IgG control: *p < 0.05; **p < 0.01. (**c**) The expression of mature human microRNAs miR-200a-3p, miR-200b-3p, miR-200b-5p, miR-200c-3p, miR-203a-3p and miR-205-5p in control (shLuc) and GRHL2-knockdown (shGRHL2 #10, #12) OVCA429 cells as analyzed by RT-qPCR. The average fold change (2^−ΔΔCt^) with respect to shLuc as control and error bars (SEM) were obtained from biological duplicates. Unpaired *t*-tests were performed on the 2^−ΔCt^ values, n.s. = not significant; *p < 0.05; **p < 0.01.

To identify microRNA (miRNA) genes directly regulated by GRHL2, we compared the levels of miRNA in OVCA429 control and shGRHL2 cells by microarray, followed by a cross-analysis with the ChIP-seq data. Interestingly, three miRNA genes that have been reported to regulate EMT—*MIR203A*, *MIR200B* and *MIR205*^41^,^42^,^43^,^44^—emerged as miRNA genes positively regulated by GRHL2, with down-regulated expression after GRHL2 knockdown (Fig. 5a). The ChIP-seq peaks for *MIR205* were found at intergenic regions or at the promoter and transcription termination site of its host gene, *MIR205HG* (Supplementary Table S3). *MIR203A* had a ChIP-seq peak at its promoter whereas *MIR200B* had one 3859 bp upstream of the hairpin-coding region, within a CpG island (Supplementary Table S3). This GRHL2 binding site of *MIR200B* was 368 bp downstream of the reported transcription start site (TSS)^41^ shared by three neighbouring genes *MIR200B, MIR200A,* and *MIR429* (Fig. 5b). Thus, GRHL2 may regulate the promoter of a polycistronic primary transcript encoding three miRNAs (miR-200b, miR-200a, and miR-429). ChIP-qPCR further confirmed the GRHL2 binding sites at the promoter region of the *MIR200B/200A/429* gene cluster and *MIR203A*, respectively (Fig. 5b). We then verified the expression changes of mature miRNAs miR-200a-3p, miR200b (5p and 3p), miR-200c-3p (another miR-200 member), miR-203a-3p and miR-205-5p in GRHL2-knockdown OVCA429 cells using RT-qPCR. All of the epithelial-related miRNAs tested were down-regulated after GRHL2 knockdown (Fig. 5c), which is in line with our miRNA microarray data (Supplementary Table S4). Hence, GRHL2 may positively regulate miRNA genes directly at the promoters (*MIR200B/200A/429* cluster and *MIR203A)* or indirectly (*MIR200C*) through other pathways.

### GRHL2 and ZEB1 form a double negative feedback regulation via miR-200b and miR-200a

**Figure 6:**
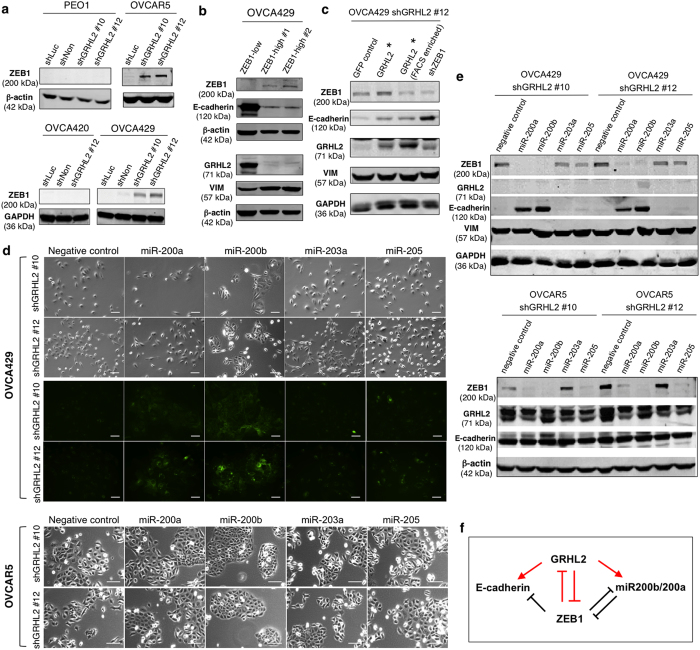
The GRHL2-ZEB1 feedback loop involves miR-200b and miR-200a. (**a**) Western blots of ZEB1 and β-actin or GAPDH in control (shLuc, shNon) and GRHL2-knockdown (shGRHL2 #10, #12) cells of PEO1, OVCA420, OVCAR5 and OVCA429. Up-regulation of ZEB1 was observed in GRHL2-knockdown OVCAR5 and OVCA429 cells. Full-length blots are presented in Supplementary Fig. S10. (**b**) Western blots of ZEB1, E-cadherin, GRHL2, vimentin and β-actin in OVCA429 control (ZEB1-low) and ZEB1-overexpressing (ZEB1-high #1, #2) cells. (**c**) Western blots of ZEB1, E-cadherin, GRHL2, vimentin and GAPDH in OVCA429 shGRHL2 #12 cells infected with GFP control, shRNA-resistant GRHL2 (before and after FACS sorting) or ZEB1-targeting shRNA. (**d**) Cell morphologies (top panel) of GRHL2-knockdown OVCA429 cells (shGRHL2 #10, #12) transfected with miRNA mimics of negative control, miR-200a, miR-200b, miR-203a or miR-205. Immunofluorescence images (middle panel) showing E-cadherin stainings (green) in OVCA429 cells transfected with miRNA mimics. Cell morphologies (bottom panel) of GRHL2-knockdown OVCAR5 cells transfected with miRNA mimics. The rescue of GRHL2-knockdown phenotype is more prominent in cells transfected with miR-200b. Scale = 100 μm. (**e**) Western blots (top) of ZEB1, GRHL2, E-cadherin, vimentin and GAPDH in GRHL2-knockdown OVCA429 cells (shGRHL2 #10, #12) transfected with miRNA mimics of negative control, miR-200a, miR-200b, miR-203a or miR-205. Western blots (bottom) of ZEB1, GRHL2, E-cadherin and β-actin in GRHL2-knockdown OVCAR5 cells transfected with miRNA mimics. Full-length blots are presented in Supplementary Fig. S10. (**f**) A proposed model of the regulatory networks involving GRHL2, E-cadherin, ZEB1 and miR-200b/a. Symbols in red represent roles of GRHL2 in EOC, as supported by the data from this study.

We compared the expression of five classical EMT-activating transcription factors—*ZEB1, ZEB2, SNAI1, SNAI2* and *TWIST1*—in control and GRHL2-knockdown cells by RT-qPCR. All five EMT drivers tested were up-regulated in GRHL2-knockdown OVCA429 cells, among which *SNAI2*, *TWIST1* and *ZEB1* showed similar increasing trends in PEO1 and OVCA420 (Supplementary Fig. S7). ZEB1 protein was significantly up-regulated after GRHL2 knockdown in both OVCA429 and OVCAR5 cells but not in PEO1 and OVCA420 cells (Fig. 6a). This suggests GRHL2 to be an upstream transcription suppressor of ZEB1, at least in the IE cell lines. Reciprocally, GRHL2 and E-cadherin protein levels were down-regulated in ZEB1-overexpressing OVCA429 cells as compared with the ZEB1-low control (Fig. 6b), suggesting that GRHL2 and ZEB1 negatively regulate each other. However, this GRHL2-ZEB1 negative feedback loop might not exist in cell lines with the E phenotype, such as PEO1 and OVCA420.

Next, we generated a GFP plasmid encoding full-length GRHL2 resistant to the shRNA #12 (designated as GRHL2*) and overexpressed it in shGRHL2 #12 OVCA429 cells. The protein level of E-cadherin was rescued partially; whereas ZEB1 was down-regulated in the GFP-positive cells enriched by FACS (Fig. 6c). Knocking down ZEB1 in shGRHL2 #12 OVCA429 cells with ZEB1-targeting shRNA resulted in a modest up-regulation of endogenous GRHL2, with a better rescue of E-cadherin expression. Despite the changes in E-cadherin and ZEB1, the protein levels of vimentin remained high in both GRHL2*-overexpressing and shZEB1 cells (Fig. 6c), with no significant reversal of cell morphology (data not shown). This suggests that the re-expression of GRHL2* or ZEB1 knockdown partially reverses the shGRHL2 #12 OVCA429 cells back to their IE state.

Transient transfection of miRNA mimics of miR-200a, miR-200b, miR-203a and miR-205 was also performed to rescue the GRHL2 knockdown phenotype in OVCA429 and OVCAR5 cells. Among the ectopically expressed miRNAs, both miR-200a and miR-200b were able to restore E-cadherin expression (Fig. 6d,e) in OVCA429, possibly through direct down-regulation of ZEB1 (Fig. 6e), as ZEB1 is known to form a double negative feedback loop with the miR-200 family^41^,^42^. Remarkably, miR-200b was able to revert the morphologies of shGRHL2 #10 and #12 cells (in both OVCA429 and OVCAR5) to an epithelial-like phenotype (Fig. 6d), suggesting it as a key target of GRHL2 in phenotype regulation. Thus, we propose that the reciprocal regulation between GRHL2 and ZEB1 involves direct transcriptional regulation of miR-200b and miR-200a (Fig. 6f). These regulatory networks between GRHL2, miR-200b/a, ZEB1 and E-cadherin are essential for phenotype maintenance in EOC cells.

### GRHL2 regulates histone modifications of *CDH1* and *MIR200B/200A/429*

**Figure 7:**
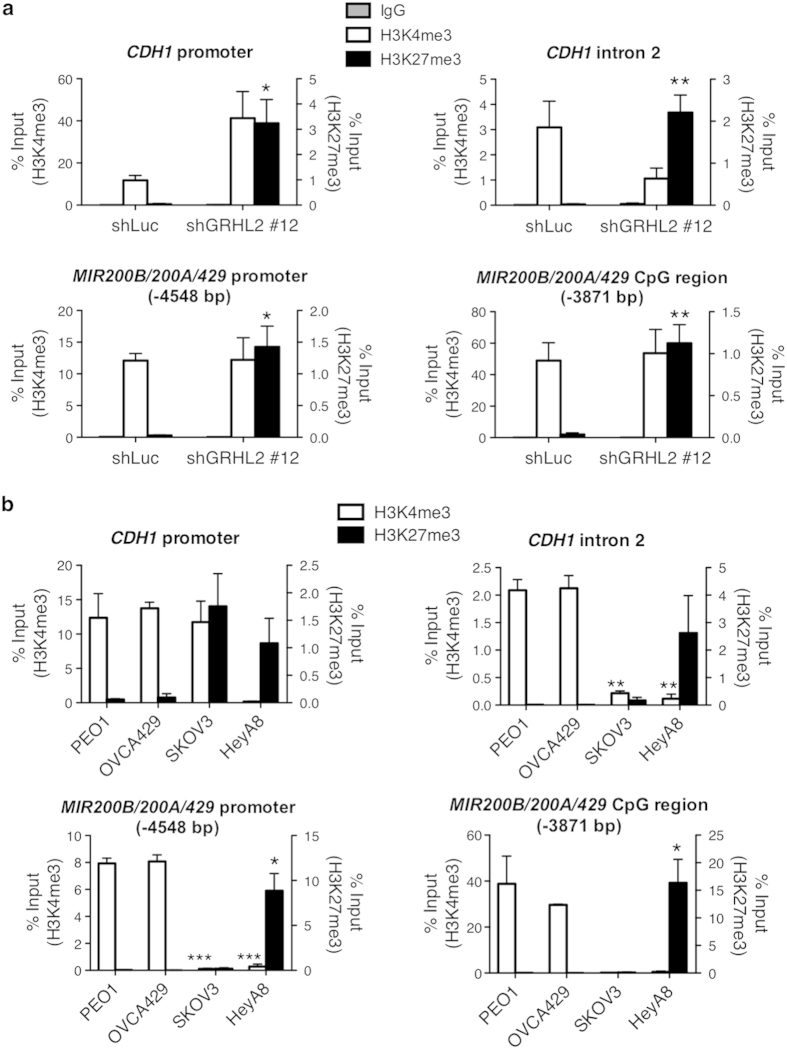
Levels of H3K4me3 and H3K27me3 along the EMT spectrum. (**a**) ChIP-qPCR of IgG control (grey, left *y*-axis), H3K4me3 (white, left *y*-axis) and H3K27me3 (black, right *y*-axis) at the promoter region and GRHL2-binding site of *CDH1* (intron 2) and *MIR200B/200A/429* cluster (CpG region) in OVCA429 shLuc and shGRHL2 #12 cells. Signals of IgG control and ChIP samples were normalized to input DNA and are presented as % input with SEM from two independent experiments. Unpaired *t*-tests comparing shGRHL2 #12 with respect to shLuc; *p < 0.05; **p < 0.01. (**b**) ChIP-qPCR results showing the levels of H3K4me3 (white, left *y*-axis) and H3K27me3 (black, right *y*-axis) at the promoter region and GRHL2-binding site of *CDH1* (intron 2) and *MIR200B/200A/429* cluster (CpG region) in PEO1, OVCA429, SKOV3 and HeyA8. Signals of IgG control and ChIP samples were normalized to input DNA and presented as % input with SEM from two independent experiments. Unpaired *t*-tests were used to compare mesenchymal-like cell lines (SKOV3 and HeyA8) with respect to epithelial-like cell lines (PEO1 and OVCA429); *p < 0.05; **p < 0.01; ***p < 0.001.

The overexpression of GRHL2* was not sufficient to fully reverse the EMT phenotype in OVCA429 shGRHL2 #12 cells. Ectopic overexpression of full-length WT GRHL2 in GRHL2-low EOC cell lines such as SKOV3, OV56 and HeyA8 resulted in neither MET-like morphological changes nor the up-regulation of E-cadherin (Supplementary Fig. S8). We hypothesized that, in shGRHL2 or GRHL2-low cells, chromatin remodeling at GRHL2 target genes could hinder the binding of ectopically expressed GRHL2. Indeed, based on our ChIP-qPCR results, the overexpressed GRHL2* in OVCA429 shGRHL2 cells showed no enrichment at the GRHL2 binding sites of its target genes (Supplementary Fig. S9). Since GRHL2 was reported to regulate histone modifications^12^,^45^, we further analyzed the levels of H3K4me3 (active chromatin) and H3K27me3 (repressed chromatin) histone marks at the promoters and GRHL2 binding sites of *CDH1* and *MIR200B/200A/429* in OVCA429 shLuc and shGRHL2 #12 cells by ChIP-qPCR. At the *CDH1* promoter, H3K4me3 remained high whereas H3K27me3 increased significantly after GRHL2 knockdown (Fig. 7a). At the GRHL2 binding site of *CDH1* (intron 2), there was a slight decrease in H3K4me3 accompanied by a significant increase in H3K27me3. For *MIR200B/200A/429*, the level of H3K4me3 remained the same, accompanied by a significant increase in H3K27me3 at both the promoter and the GRHL2 binding site (CpG region) after GRHL2 knockdown. This consistent increment in H3K27me3 repressive mark may have contributed to the down-regulation of E-cadherin and miR-200b in shGRHL2 #12 cells.

A similar ChIP-qPCR was performed using PEO1, OVCA429, SKOV3, and HeyA8 cells representing the E, IE, IM, and M phenotypes. Along this EMT spectrum, the levels of H3K4me3 in PEO1, OVCA429 and SKOV3 remained high at the *CDH1* promoter but dropped drastically in HeyA8 of the M phenotype (Fig. 7b). An increase of H3K27me3 was observed in both SKOV3 and HeyA8. At *CDH1* intron 2, the levels of H3K4me3 were significantly lower in both SKOV3 and HeyA8 as compared with that in PEO1 and OVCA429, and accompanied by higher levels of H3K27me3, especially in HeyA8. A similar trend was observed at both the promoter and the CpG region of *MIR200B/200A/429*. Hence, our data indicate a switch between the active and repressive histone modifications following the loss of GRHL2, at both the promoters and GRHL2 binding sites of its epithelial target genes *CDH1* and *MIR200A/200B/429*. These results suggest that, in addition to transcriptional modulators, other regulatory barriers controlled by epigenetics are present, which hinder the phenotype reversal in EOC cells with a mesenchymal-like phenotype and might explain the partial rescue attained by GRHL2*.

## Discussion

*GRHL2* is overexpressed and amplified in various cancers^18^,^26^,^27^. In this study, we scrutinized the expression of *GRHL2*, within and across different cancer types, in relation to the EMT score. With its expression negatively correlated with the EMT score, *GRHL2* serves as an epithelial signature in cancers. Interestingly, although the median *GRHL2* expression displayed a bimodal-like distribution, with an almost equally low *GRHL2* expression in cancers with high EMT scores, we observed a continuous increase of EMT scores in cancers with low GRHL2 expression. This suggests *GRHL2* to be a robust indicator of the epithelial trait but that its loss alone may not be sufficient to effect a mesenchymal trait. This is in line with our observation that knocking down GRHL2 was unable to overcome the anchorage dependency in EOC.

Our results showed that knockdown of GRHL2 in EOC cells led to a partial or full EMT. Hence, GRHL2 could be a gatekeeper for sequential EMT execution. This is consistent with findings in breast cancer that implicate GRHL2 as an EMT suppressor, with significant lower expression in the claudin-low subtype^19^,^21^. The flattened morphology observed in shGRHL2 #10 EOC cells also matches the cell-flattening events reported in alveolar epithelial cells after the down-regulation of GRHL2 during epithelial injury, which may lead to idiopathic pulmonary fibrosis^15^,^46^. However, the extent of this EMT execution is context-dependent. Although EOC cells from both E (OVCAR3 and PEO1) and IE (OVCA429) phenotypes showed GRHL2 binding at the intron 2 enhancer of *CDH1*, down-regulation of E-cadherin (both mRNA and protein) was only observed in OVCA429 after GRHL2 knockdown. It remains unclear whether this was due to incomplete silencing of GRHL2 by the shRNAs or the inherent differences in epigenetic controls among the cell lines. Furthermore, up-regulation of ZEB1 after GRHL2 knockdown was evident in both OVCA429 and OVCAR5 but not in PEO1 or OVCA420. We speculate that epithelial-like EOC cell lines such as PEO1 and OVCA420 might have a tighter transcriptional control over epithelial genes and, hence, are less susceptible to phenotypic changes mediated by GRHL2 knockdown; OVCA429, by contrast, already has an intermediate phenotype with a considerable level of plasticity. This supports the existence of an EMT spectrum in EOC^9^, within which, cells with an IE phenotype are likely to have higher EMT potential than cells with an E phenotype.

In breast cancer, GRHL2 has been reported to suppress anoikis resistance and mammosphere formation^19^. However, GRHL2 knockdown did not improve the anchorage-independent growth of EOC cells. Furthermore, OVCA429 shGRHL2 #10 cells showed slower growth as compared with the control cells under normal culture conditions. Hence, the role of GRHL2 as a tumour suppressor in EOC can only be surmised from the positive correlation of GRHL2 expression with a better survival. Indeed, the function of GRHL2 in tumourigenesis has remained contradictory. In breast cancer, Cieply *et al.* suggested GRHL2 to be a tumour suppressor that inhibits tumour initiation, promotes chemosensitivity, and suppresses stem cell characteristics^20^. However, other studies have implicated GRHL2 as an oncogene that promotes tumour growth and metastasis^21^,^22^,^23^,^27^,^47^. In HCC^26^, OSCC^18^, and colorectal carcinoma^25^, GRHL2 is reported to be oncogenic, possibly via the regulation of telomerase activity^18^. This contradicts the findings in gastric cancer that GRHL2 suppresses tumour growth by inducing apoptosis and inhibiting cell proliferation^24^. The role of GRHL2 in regulating tumour growth requires further clarification.

GRHL2 and ZEB1 have been shown to directly repress each other^19^,^20^,^21^,^25^. However, in EOC, no significant binding of GRHL2 has been found at the promoter of *ZEB1*. Only one GRHL2 binding site was detected in OVCA429 cells, within the intron 1 of the *ZEB1* gene. Therefore, GRHL2 may not directly repress the promoter activity of *ZEB1* in EOC cells. Our findings suggest that the reciprocal repressive relationship between GRHL2 and ZEB1 may exist through the miR-200 members. These miRNAs have been shown to suppress EMT by repressing *ZEB1* and *ZEB2* at the 3′UTR^42^. Reciprocally, ZEB1 binds to and represses the promoter of the three co-regulated *MIR200B/200A/*429^41^, thus forming a double negative feedback regulation with the three miR-200 members, exerting an opposite influence on EMT^44^. We showed that miR-200a, miR-200b and miR-200c were down-regulated after GRHL2 knockdown, and that the ectopic expression of either miR-200a or miR-200b was sufficient to rescue E-cadherin expression in shGRHL2 cells. Furthermore, miR-200b induced a MET-like morphological change. Therefore, our data support a pivotal role of GRHL2 in the regulation of epithelial-specific miRNAs and their respective targets.

Despite being in the same family, the five members of miR-200 are divided into two functional groups (miR-200b/200c/429 and miR-200a/141) based on their seed regions that differ at one position from the other, and each group could target different or the same sets of genes^48^. It has been shown that the miR-200b/200c/429 but not the miR-141/200a functional group alters cell morphology and represses metastasis of breast cancer cells in mouse xenografts, via the regulation of genes involved in cytoskeletal remodelling, independently of the ZEB1-E-cadherin pathway^49^. In addition, miR-200b but not miR-200a has been shown to decrease Rho activity in breast cancer cells^48^. These findings may explain that although both miR-200a and miR-200b were able to restore E-cadherin expression in EOC cells, only miR-200b-transfected shGRHL2 cells could undergo a strong MET-like morphological reversal. Similarly, even though E-cadherin levels were unaffected after GRHL2 knockdown, changes in cell morphology were evident in OVCA420 and OVCAR5 cells, which showed altered subcellular localization of E-cadherin. Therefore, alterations in E-cadherin levels, a well-accepted indicator of phenotype switch, may not necessarily precede changes in cell morphology or cell behavior that involves cytoskeletal remodeling. It will be interesting to further examine the target genes of GRHL2 or miR-200b involved in actomyosin contractility and cytoskeletal organization during early or partial phenotype transitions.

In mouse kidney cells, the loss of Grhl2 results in reduced active histone marks (H3K4me3and H3-K9/14ac) at the *CDH1* promoter^12^. In keratinocytes, GRHL2 has been shown to inhibit the recruitment of histone demethylase JMJD3, resulting in elevated levels of H3K27me3 at the promoters of its target genes^45^. In EOC, we showed that GRHL2 knockdown significantly increased the H3K27me3 levels at the promoters and GRHL2 binding sites of *CDH1* and *MIR200B/200A/429.* Thus, besides transcriptional regulation, GRHL2 may control the expression of its target genes through histone modifications such as H3K27me3. GRHL2 has also been reported to inhibit DNA methylation at the 5′ CpG island of telomerase gene (*TERT)*, possibly by hindering the activity of DNA methyltransferase DNMT1^38^. GRHL2 itself may also be regulated by DNA methylation^50^ or histone modifications. We showed that within a heterogeneous cancer type such as EOC, different patterns of histone modifications exist along the EMT spectrum. Hence, the interplay between EMT transcription factors and epigenetic modifications may shed light on the regulation of EMT.

## Materials and Methods

### Cell culture

42 EOC cell lines from the SGOCL cell line library^9^,^51^ were used in this study. The cell lines were maintained in different media (Supplementary Table S5).

### Patient samples

A collection of archived frozen EOC patient samples were obtained from the Department of Gynecology & Obstetrics, Kyoto University Graduate School of Medicine, Japan and the Department of Obstetrics & Gynecology, Tri-Service General Hospital, Taiwan^52^. From the collection, 44 samples were previously subclassified into different molecular subgroups^32^ (Supplementary Table S6). RNAs from these samples were used for RT-qPCR.

### Generation of stable cell lines and transient transfectants

GRHL2-targeting shRNA (TRCN0000015810 and TRCN0000015812) and negative control plasmids—Luciferase shRNA and non-targeting shRNA (SHC007, SHC016) were purchased from Sigma. Plasmids were incubated with Lentiviral Packaging Mix (SHP001, Sigma) and Fugene 6 (Roche) before addition to 293T cells. Viral supernatants were harvested to infect cells with the addition of 8 μg/ml polybrene (Sigma). After 48 h, infected cells were selected by puromycin (4 to 7 μg/ml). For ZEB1 overexpression, plasmid pCMV6-AC-GFP-ZEB1 generated from pCMV6-Entry-ZEB1 (RC217704, Origene) was used. Transfected cells were selected by G418 (Life Technologies) at 300 μg/ml, and three single clones (one ZEB1-low, two ZEB1-high) were picked. For GRHL2 overexpression, pLenti-GIII-CMV-GFP-2A-Puro was purchased from Applied Biological Materials. shRNA-resistant GRHL2* with four silent mutations was generated using Quick Change II XL Site-Directed Mutagenesis kit (Stratagene). For overexpression of mature miRNAs, microRNA mimics (HMC0002, HMI0357, HMI0350, HMI0352, HMI0359; Sigma) were transfected into cells by HiPerfect® transfection reagent (Qiagen).

### RT-qPCR

Total RNA of EOC cell lines and EOC tumour samples was extracted with miRNeasy mini kit (Qiagen). 500ng RNA was reverse-transcribed using RT^2^ First Strand kit (SAbiosciences, Qiagen) and mixed with SYBR green master mix (SAbiosciences, Qiagen) for qPCR using ABI 7900HT (Life Technologies). Primers were purchased from SAbiosciences (Qiagen) and at least three out of the five housekeeping genes (*B2M, HPRT1, RPL13A, GAPDH* and *ACTB*) were used for normalization. The mRNA expression level of each gene was normalized to the expression of housekeeping genes and presented either as average 2^−∆Ct^, or as average fold change (2^−∆∆Ct^) with respect to control, from at least two biological replicates. For quantification of miRNA, 500 ng of total RNA was reverse transcribed using miScript II RT kit (Qiagen). qPCR was performed using miScript SYBR Green PCR kit (Qiagen) and ABI 7900HT. *RNU6-6P* was used as an internal control. All primers used (Qiagen) are listed in Supplementary Table S7.

### Western blot analysis

Cell lysates were resolved by reducing SDS-PAGE, and assessed by standard western blotting. Antibodies used included: anti-GRHL2 (HPA004820) from Sigma-Aldrich; anti-E-cadherin (610182) from BD Transduction Laboratories; anti-ZEB1 (3396) and anti-ErbB3 (12708) from Cell Signaling Technology; anti-EPCAM/TROP1 (MAB960) and anti-TACSTD2/TROP2 (AF650) from R&D Systems; anti-Vimentin (M7020) from Dako; anti-β-actin (A1978) and anti-GAPDH (G9545) from Sigma-Aldrich. Secondary antibodies from Li-COR Biosciences were used: IRDye 800CW goat anti-mouse/rabbit (926-32210, 926-32211), IRDye 680LT goat anti-mouse/rabbit (926-68020, 926-68021) and IRDye 800CW donkey anti-goat (926-32214). Blots were scanned using the Odyssey Infrared Imaging System (Li-COR).

### Immunofluorescence staining

Cells grown on glass coverslips were fixed with 4% paraformaldehyde for 10 minutes, and treated with 0.05% Triton-X for 5 min. For immunostaining, fixed cells were incubated with blocking buffer (3% BSA in 1× PBS) for 1 h and then stained with primary antibodies for 1 h at 37 °C. Antibodies used included: anti-GRHL2 (HPA004820, Sigma-Aldrich); anti-E-cadherin (610182, BD); anti-N-cadherin (M142, Takara); anti-pan-Cytokeratin (M3515) and anti-Vimentin (M7020) from Dako; anti-β-Catenin (8480), anti-phospho-Myosin Light Chain 2 (Ser19) (3671), anti-LC3A (4599), anti-EEA1 (3288), anti-LAMP1 (9091), anti-RCAS1 (12290) from Cell Signaling Technology. Alexa Fluor 488-conjugated anti-mouse/rabbit (A11029/A11034) and Alexa Fluor 594-conjugated anti-mouse/rabbit (A11032/A11037) from Invitrogen were used as secondary antibodies. Rhodamine phalloidin probe (R415, Life Technologies) was used for F-actin staining. Cover slips were mounted onto glass slides using Vectashield mounting medium with/without DAPI (H-1200, H-1000) from Vector Laboratories. Images were taken using a Nikon A1R confocal system or a Zeiss AxioImager M2 epifluorescence imaging system.

### Gap closure migration assay, invasion assay and live-cell imaging

For the gap closure migration assay, cells were seeded into culture inserts (80209, Ibidi) and grown until 90% to 100% confluence. The cells were then subjected to serum starvation before the inserts were removed. Non-attached cells were washed away and fresh L-15 media (21083-027, Life Technologies) supplemented with 20% FBS, was added to the cells for time-lapse microscopy using a Zeiss Axio Observer Z1 Live-imaging System with at 37 °C incubation. For the invasion assay, 300 μl of pre-chilled BD Matrigel (354234, BD BioSciences), at a concentration of 8.9 mg/ml, was layered on top of the attached cells. The gel was allowed to solidify by incubation at 37 °C for 1 h. Prior to live imaging, 1 ml of L-15 media supplemented with 20% FBS was added. Wimscratch (Wimasis) and ImageJ (NIH) were used for image analysis.

### Quantitative ELISA

MMP2 in cultured media was quantified using Quantikine ELISA kit (DMP2F0, R&D). Cells were seeded onto 60-mm dishes overnight, and then incubated with fresh media at 37 °C, 5% CO_2_ for 24 h. Cultured supernatants were harvested, filtered using a 20-μm filter. Undiluted samples (100 μl) were used for the immunoassay according to the manufacturer’s protocol. Absorbance signals were detected using a microplate reader (Tecan Infinite 200).

### ChIP-qPCR and ChIP-seq

Cells were cross-linked using 1% formaldehyde for 10 min and then treated with 0.125 M glycine for 5 min. The fixed cells were rinsed with 1× TBS and harvested by scraping in SDS buffer (100 mM NaCl, 50 mM Tris-Cl, pH 8.1, 5 mM EDTA, 0.5% SDS, 0.02% NaN_3_ and protease inhibitors). Cells were sonicated on ice to achieve chromatin sizes of 200 bp to 500 bp. Samples were incubated with IgG, anti-GRHL2 (HPA004820, Sigma), anti-H3K4me3 (CS-003-100, Diagenode) or anti-H3K27me3 (07-449, Millipore) antibodies overnight at 4 °C. Protein G sepharose beads (17-0618-02, GE Healthcare) were added and incubated for 2 h at 4 °C, followed by high stringency washes. Bound DNA was eluted, reverse cross-linked and purified using the QIAquick PCR purification kit (Qiagen). Purified samples and input controls were used for qPCR or next-generation sequencing. Primers used are listed in Supplementary Table S8. ChIP-seq library was constructed using NEBNext^®^ ChIP-seq sample preparation kit (E6200, New England Biolabs) according to the manufacturer’s protocol. Size-selection was performed by gel extraction after agarose gel electrophoresis. Multiplexing sample preparation oligonucleotide kit (PE-400-1001, Illumina) was used for index labelling.

### ChIP-seq data analysis

At least 16 million 51-bp long reads were mapped to hg19 using bowtie v2.1.0^53^ with parameters -N 1 –sensitive -p 2 –no-unal. Peaks were identified by MACS 2.0.9^36^ using a maximum of 2 reads per unique position and otherwise default parameters. Only highly enriched (enrichment over background ≥5-fold, pileup ≥25) and highly significant (*q*-value < 0.01) peaks were shortlisted from the analysis, which yielded 1549 peaks in OVCAR3, 6589 peaks in PEO1 and 9412 peaks in OVCAR429. *de novo* Motif search was conducted by MEME-ChIP^54^ using the peak center extended by 75 bp in both directions. The distribution of GRHL2 binding sites with reference to hg19 annotations was analyzed using HOMER (Hypergeometric Optimization of Motif EnRichment)^55^ v4.5.

### Microarray gene expression analysis

Total RNA was extracted with miRNeasy mini kit (Qiagen). RNA quality was checked on an Agilent Bioanalyzer to ensure a RIN value >7. GeneChip® Human Gene 2.0 ST Array and GeneChip® miRNA 2.0 Array (Affymetrix) were used for gene expression and miRNA analyses (Origen Laboratories). Microarray data were pre-processed and Robust Multi-array Average (RMA)-normalized using Affymetrix Power Tools 1.15.2 and annotation files version na.33.2. Probes that were detected significantly above background (DABG, *p* < 0.05) in at least one sample were retained for further analysis. The gene expression levels of each transcript were averaged across exons based on the metaprobesets version r4 annotation from Affymetrix.

### Accession number

The original ChIP-seq and microarray data have been deposited in the NCBI’s Sequence Read Archive with the GEO accession number GSE71019.

### Statistics

Overall patient survival for *GRHL2*-high and *GRHL2*-low tumours were compared using a log-rank test. Two-way ANOVA and Bonferroni *post*-*hoc* tests were used to analyze differences in percentages of cell-covered areas in the gap closure assays. For other experiments, differences between control and experimental mean values were analyzed using unpaired Student’s *t-*tests, unless otherwise stated.

## Additional Information

**How to cite this article**: Chung, V. Y. *et al.* GRHL2-miR-200-ZEB1 maintains the epithelial status of ovarian cancer through transcriptional regulation and histone modification. *Sci. Rep.* **6**, 19943; doi: 10.1038/srep19943 (2016).

## Supplementary Material

Supplementary Information
