# Supplementary material for: GRHL2-miR-200-ZEB1 maintains the epithelial status of ovarian cancer through transcriptional regulation and histone modification
Source: Sci Rep. 2016 Feb 18;6:19943. doi: 10.1038/srep19943 (PMC4757891; doi:10.1038/srep19943)
Supplement: Supplementary Information [file srep19943-s1.pdf]

**GRHL2-miR-200-ZEB1 maintains the epithelial status of ovarian cancer through transcriptional regulation and histone modification.**

Vin Yee Chung[1], Tuan Zea Tan[1], Ming Tan[1], Meng Kang Wong[1], Kuee Theng Kuay[1], Zhe Yang[1], Jieru Ye[1], Julius Muller[2], Cheryl Mei-Yi Koh[2], Ernesto Guccione[2], Jean Paul Thiery[1,2,3], Ruby Yun-Ju Huang[1,4,5,*]

[1]Cancer Science Institute of Singapore, National University of Singapore;

[2]Institute of Molecular and Cell Biology, A*STAR;

[3]Department of Biochemistry, Yong Loo Lin School of Medicine, National University of Singapore;

[4]Department of Obstetrics and Gynecology, National University Hospital;

[5]Department of Anatomy, Yong Loo Lin School of Medicine, National University of Singapore.

* Corresponding author: Ruby Yun-Ju Huang, email: ruby_yj_huang@nuhs.edu.sg

## Supplementary Results

### GRHL2 knockdown up-regulates N-cadherin expression and does not affect cytokeratin expression

For mesenchymal markers, such as N-cadherin (*CDH2*) and vimentin (*VIM*), the mRNA expression was up-regulated after GRHL2 knockdown, especially in OVCA429 cells (Supplementary Figure S2a). For N-cadherin, the increment in protein expression was observed only in the OVCA429 shGRHL2 #10 cells (Supplementary Figure S2c). Cytokeratins were present in all the control and GRHL2-knockdown cells tested, as shown by immunofluorescence stainings (Supplementary Figure S2d).

## Supplementary Methods

### Anoikis assay

Anoikis resistance of control and GRHL2-knockdown OVCA429 cells was tested by seeding 3000, 6000 or 12,000 cells into each well of a 96-well plate of ultra-low attachment grade (3474, Corning). Cells were incubated at 37°C in 5% $CO_2$, and MTS assays (G5430, Promega) were performed at day 0, day 2, day 4 and day 6 according to a standard protocol. The cells were incubated with MTS reagent mix for 2 h and absorbance was read using a microplate reader (Tecan Infinite 200).

### Spheroid invasion assay

Control and GRHL2-knockdown OVCA429 cells were grown as spheroids following the protocol of a 96-well 3D Spheroid BME Cell Invasion Assay from Cultrex (3500-096-K), with modifications. Approximately 5000 cells were mixed with Spheroid Formation ECM and seeded into each well of a round-bottom, ultra-

low attachment, 96-well plate (7007, Corning). The plate was centrifuged at 200 ×*g* for 3 min and incubated at 37°C for 3 days to induce spheroid growth. After the assembled cells formed spheroids, BME matrix (Basement Membrane Extract) was added to each well (50 µl) and the plate centrifuged at 300 × *g* at 4°C for 5 min. The plate was incubated at 37°C for 1 h to allow for gel formation, before the addition of 100 µl of cell culture media. The spheroids were grown under normal cell culture condition (37°C, 5% $CO_2$) and were monitored every 3 or 4 days. Cells were stained for 1 h with Calcein AM (C3100MP, Life Technologies) to check for viability, and with Ethidium Homodimer-1 (E1169, Life Technologies) to check for dead cells. Images were taken using a Zeiss AxioImager M2 epifluorescence imaging system.

## Supplementary Figures

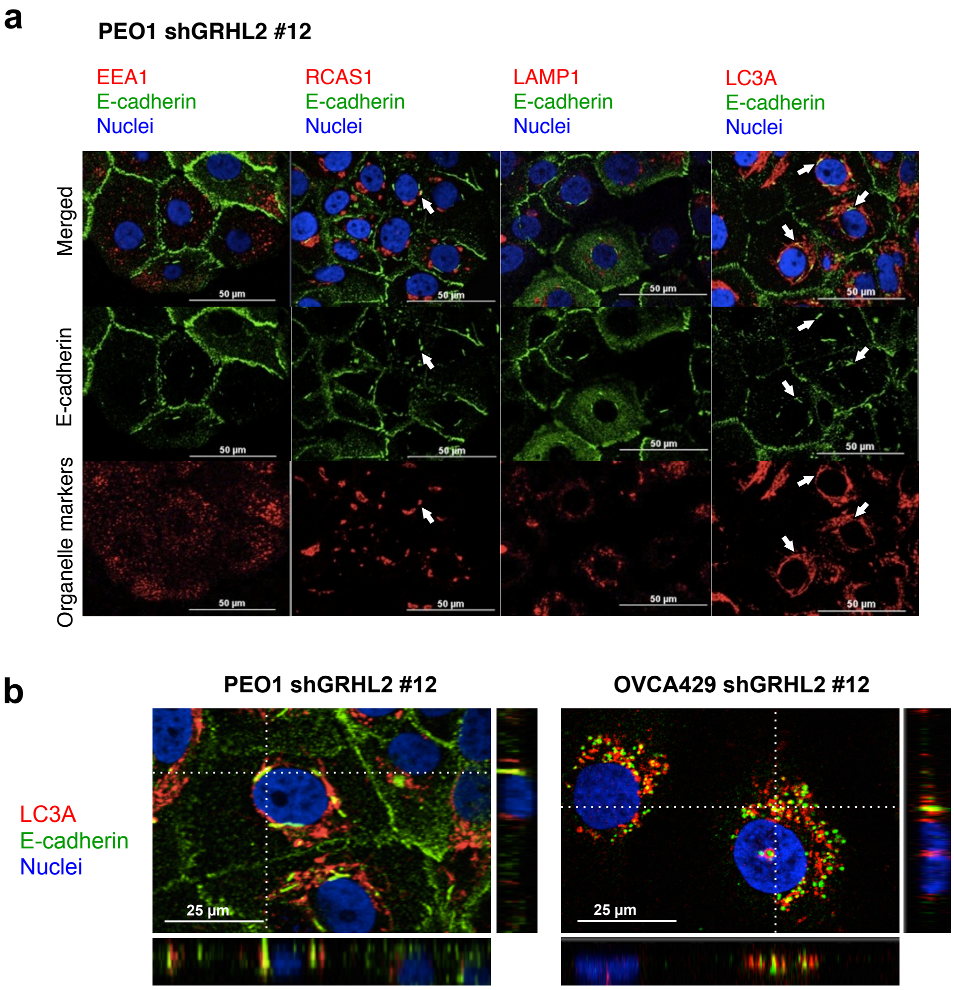


**Supplementary Figure S1. Co-localization of cytoplasmic E-cadherin with various organelle markers in GRHL2-knockdown EOC cells.**
(**a**) Immunofluorescence stainings of E-cadherin (green) and four organelle markers (red) in PEO1 shGRHL2 #12 cells. EEA1 (far left) marks the early endosome; RCAS1 (middle left) (also known as EBAG9) marks the Golgi apparatus; LAMP1 (middle right) marks lysosomes; LC3A (far right) marks autophagosomes. White arrows indicate co-localization (yellow). Scale = 50 µm. (**b**) Z-stack slices showing the co-localization (yellow) of LC3A (red) and cytoplasmic E-cadherin (green) in GRHL2-knockdown cells of PEO1 and OVCA429. Scale = 25 µm.

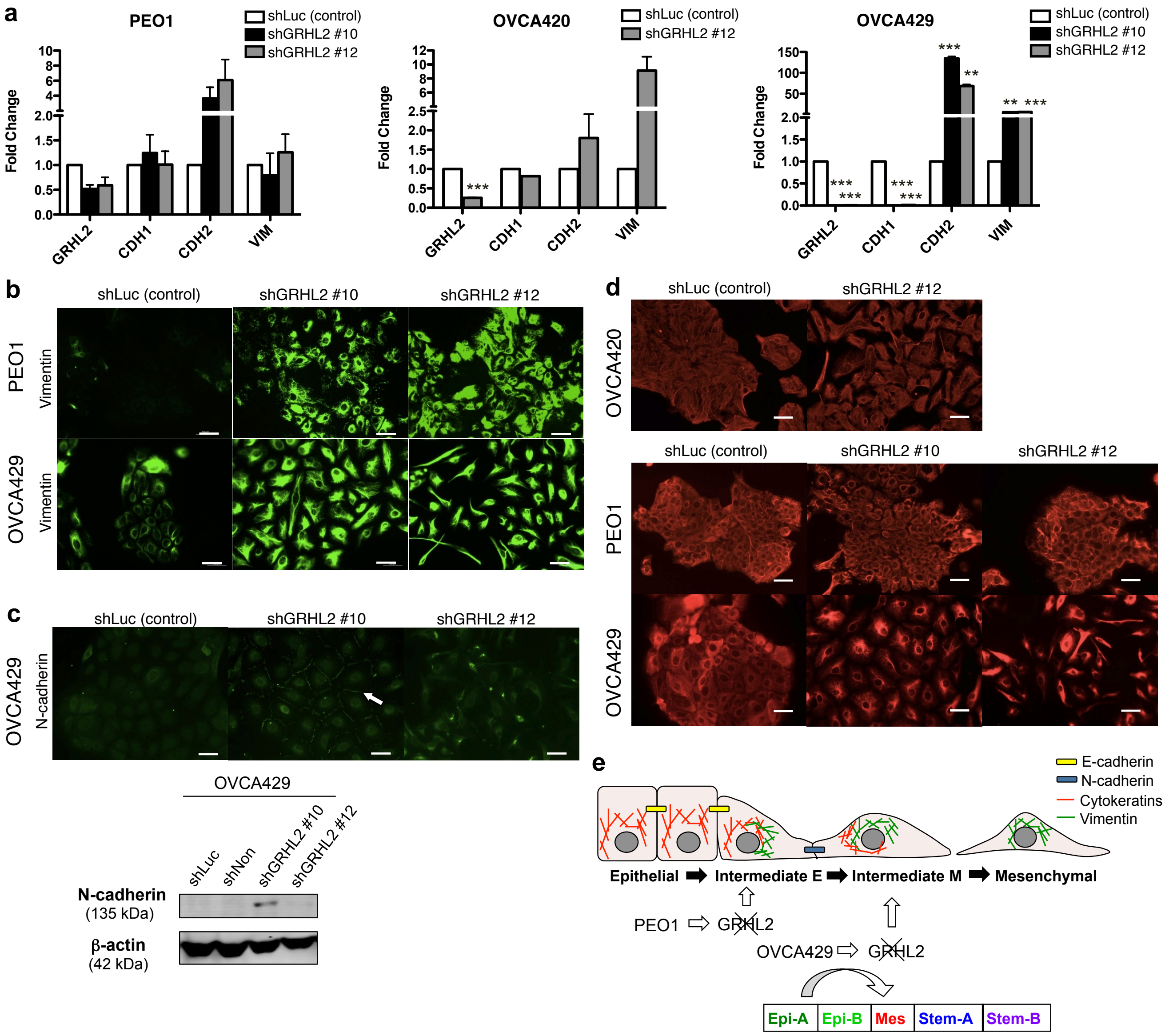


**Supplementary Figure S2. Knockdown of GRHL2 results in the up-regulation of vimentin and N-cadherin.**

(**a**) mRNA expression fold change (y-axis) in GRHL2, E- cadherin (*CDH1*), N-cadherin (*CDH2*) and vimentin (*VIM*), as measured by RT-qPCR in shLuc control (white bars), shGRHL2 #10 (black bars) and shGRHL2 #12 (grey bars) cells of PEO1, OVCA420 and OVCA429. Unpaired *t*-tests were performed on the $2^{-\Delta Ct}$ values for each gene, ** $p < 0.01$; *** $p < 0.001$. (**b**) Immunofluorescence stainings of vimentin in control (shLuc, shNon) and GRHL2-knockdown (shGRHL2 #10, #12) cells of PEO1 and OVCA429. Scale = 50 µm. (**c**) Immunofluorescence stainings (upper) of N-cadherin in control (shLuc, shNon) and GRHL2-knockdown (shGRHL2 #10, #12) OVCA429 cells. White arrow indicates junctional N-cadherin observed in shGRHL2 #10 OVCA429 cells. Scale = 50 µm. Western blots (lower) of N-cadherin and β-actin in the stable cell lines of OVCA429. Full-length blots are presented in Supplementary Figure S10. (**d**) Immunofluorescence stainings of pan-cytokeratin in the stable cell lines of PEO1, OVCA420 and OVCA429. Scale = 50 µm. (**e**) A simplified representation of the stepwise changes that occur in the phenotype (PEO1, OVCA429) and molecular subtype (OVCA429) of GRHL2-knockdown cells involving EMT markers E-cadherin, N-cadherin, cytokeratin and vimentin.

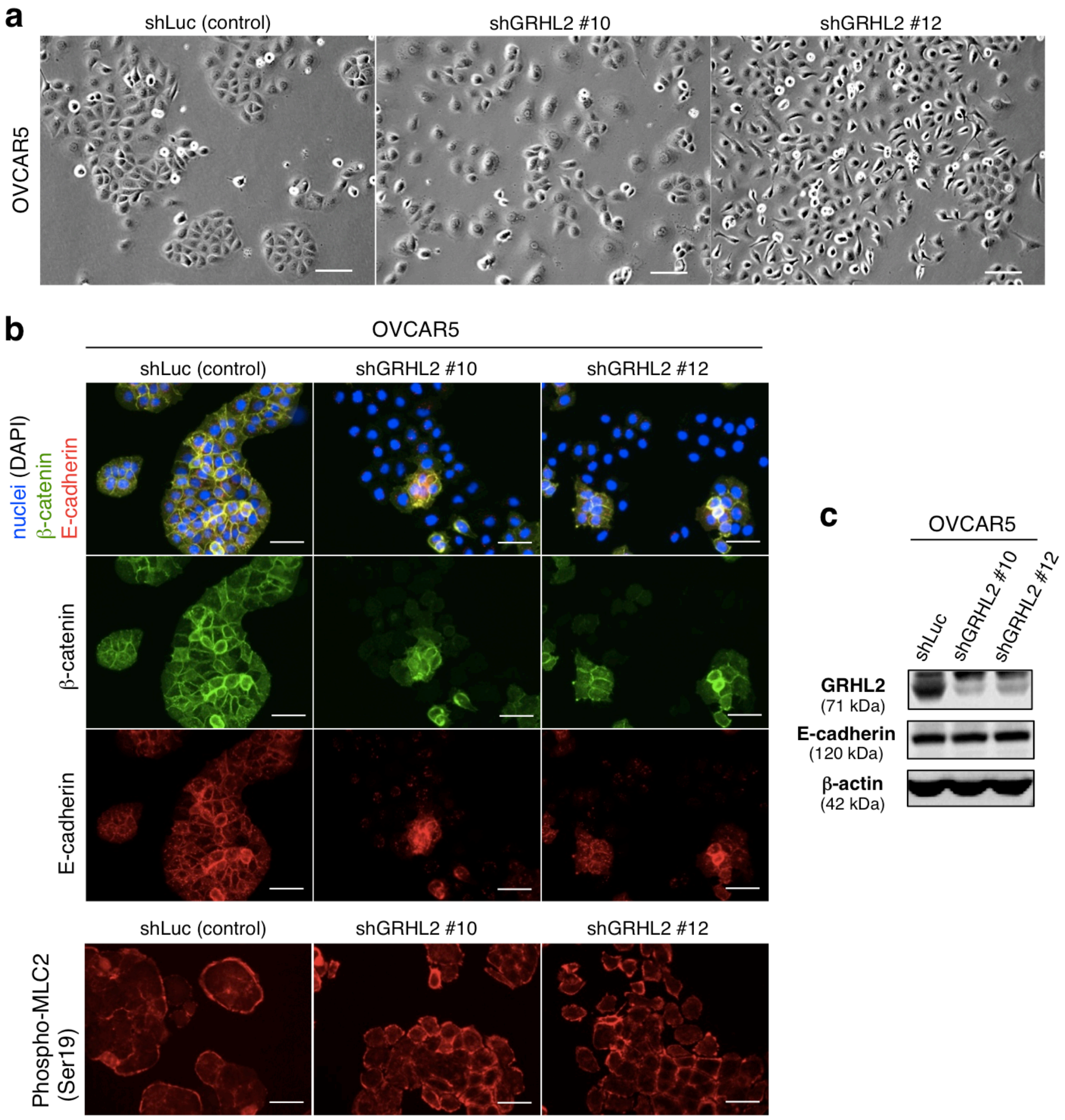


**Supplementary Figure S3. Effects of GRHL2 knockdown in OVCAR5 cells.**
(**a**) The morphologies of OVCAR5 cells infected with control shRNA (shLuc) and GRHL2-targeting shRNAs (shGRHL2 #10, shGRHL2 #12). Scale = 100 μm. (**b**) Immunofluorescence stainings of E-cadherin (red) and β-catenin (green) in the infected OVCAR5 cells. Nuclei were stained blue (DAPI). Immunofluorescence stainings (bottom) of phosphorylated myosin light chain 2 (ser19) (phospho-MLC2) in OVCAR5 cells. Scale = 50 μm. (**c**) Western blots of GRHL2, E-cadherin and β-actin in OVCAR5 cells. Upper bands in the GRHL2 panel were deemed non-specific. Full-length blots are presented in Supplementary Figure S10.

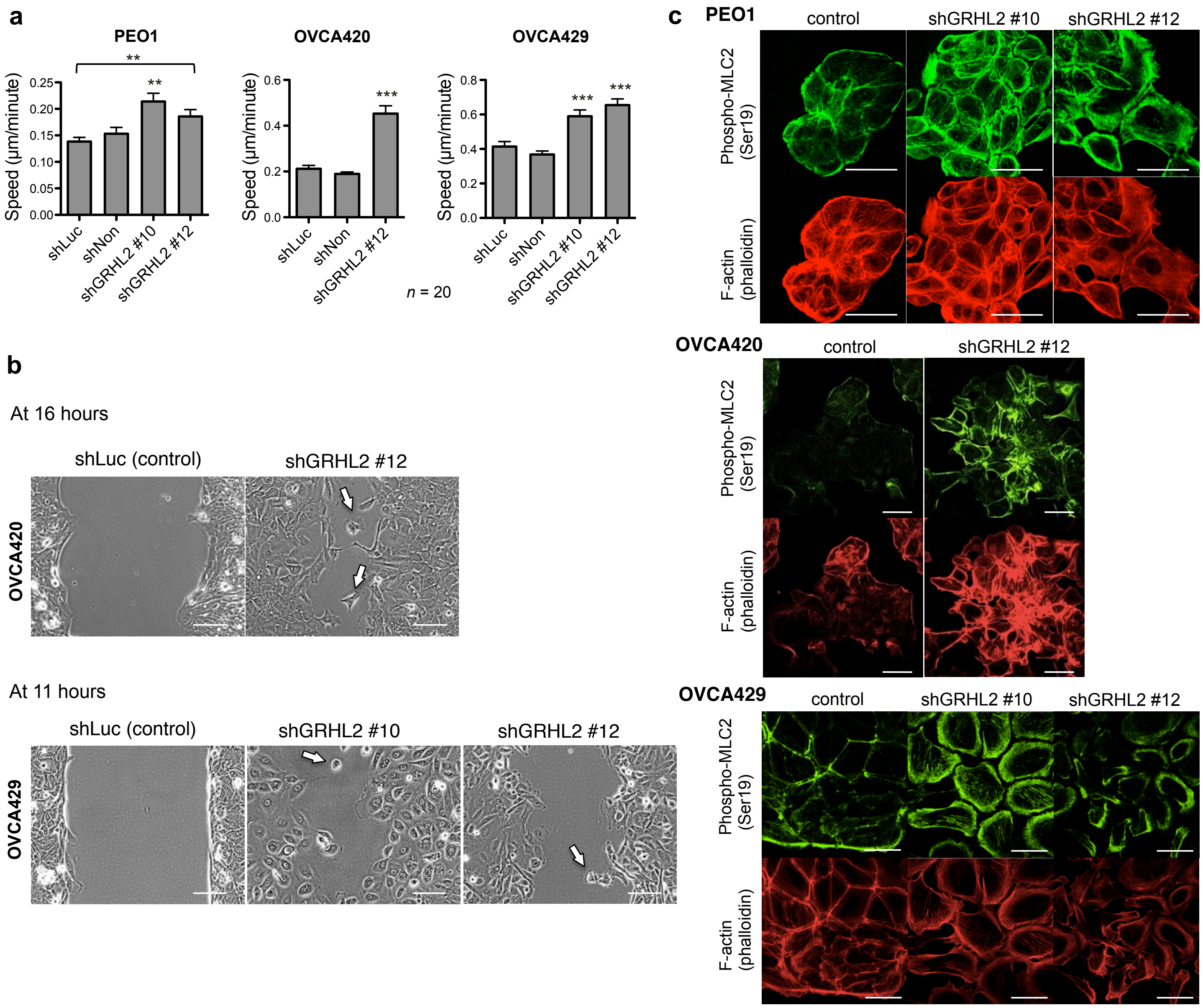


**Supplementary Figure S4. Knockdown of GRHL2 promotes cell motility and scattering in EOC.**

(**a**) The motility of control and GRHL2-knockdown PEO1, OVCA420, and OVCA429 cells as observed by time-lapse imaging ($n = 20$). Bar charts show the average speed of cell locomotion over the course of 11 to 18 h. Unpaired *t*-tests. ** $p < 0.01$; *** $p < 0.001$. (**b**) Phase-contrast images showing the migrating front of control and GRHL2-knockdown OVCA420 and OVCA429 cells during gap closure. Arrows indicate scattered, detached cells. Scale = 100 µm. (**c**) Immunofluorescence images showing phosphorylation of myosin light chain 2 (ser19) (phospho-MLC2) and F-actin (phalloidin) staining in control and GRHL2-knockdown PEO1 (upper panels), OVCA420 (middle panels), and OVCA429 (lower panels) cells. Scale = 50 µm.

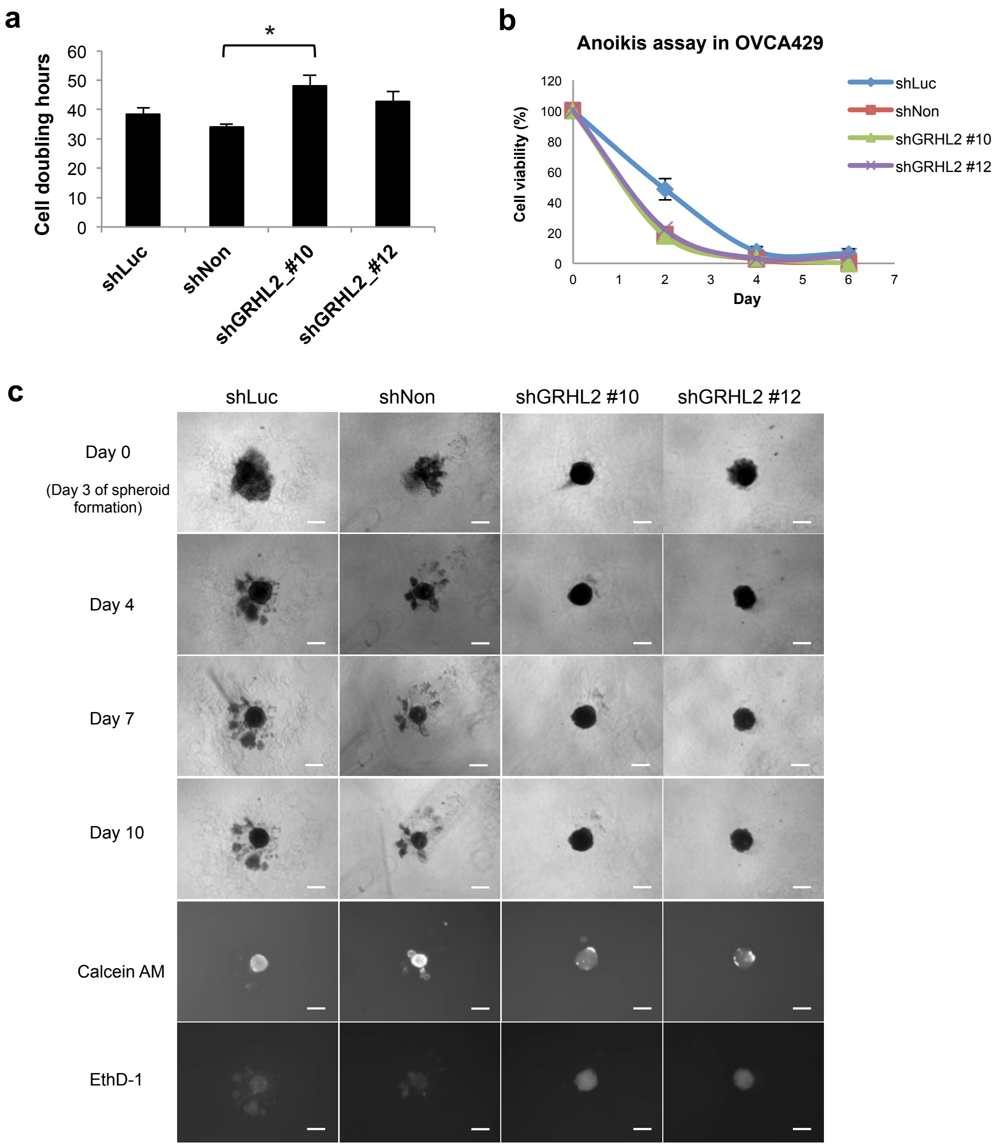


**Supplementary Figure S5. Effects of GRHL2 knockdown on cell doubling, anoikis and spheroid growth.**

(**a**) Bar charts showing the doubling hours of control (shLuc, shNon) and GRHL2-knockdown (shGRHL2 #10, #12) OVCA429 cells. Statistical significance was determined by unpaired *t*-tests. * $p < 0.05$. (**b**) Line graph showing the anoikis resistance of OVCA429 stable cell lines grown on ultra-low attachment grade culture plates, presented as the percentage of viability (*y*-axis) examined by MTS assays, at Days 0, 2, 4 and 6 (*x*-axis). (**c**) Phase contrast images of OVCA429 stable cell lines grown as spheroids for three days on ultra-low attachment culture plates (Day 0) and subsequently embedded in basement membrane extract. Serial images of spheroids at Days 4, 7, and 10 are shown. At Day 15, the spheroids were stained with calcein AM to detect viable cells and ethidium homodimer-1 (EthD-1) to label dying/dead cells. Scale = 200 μm.

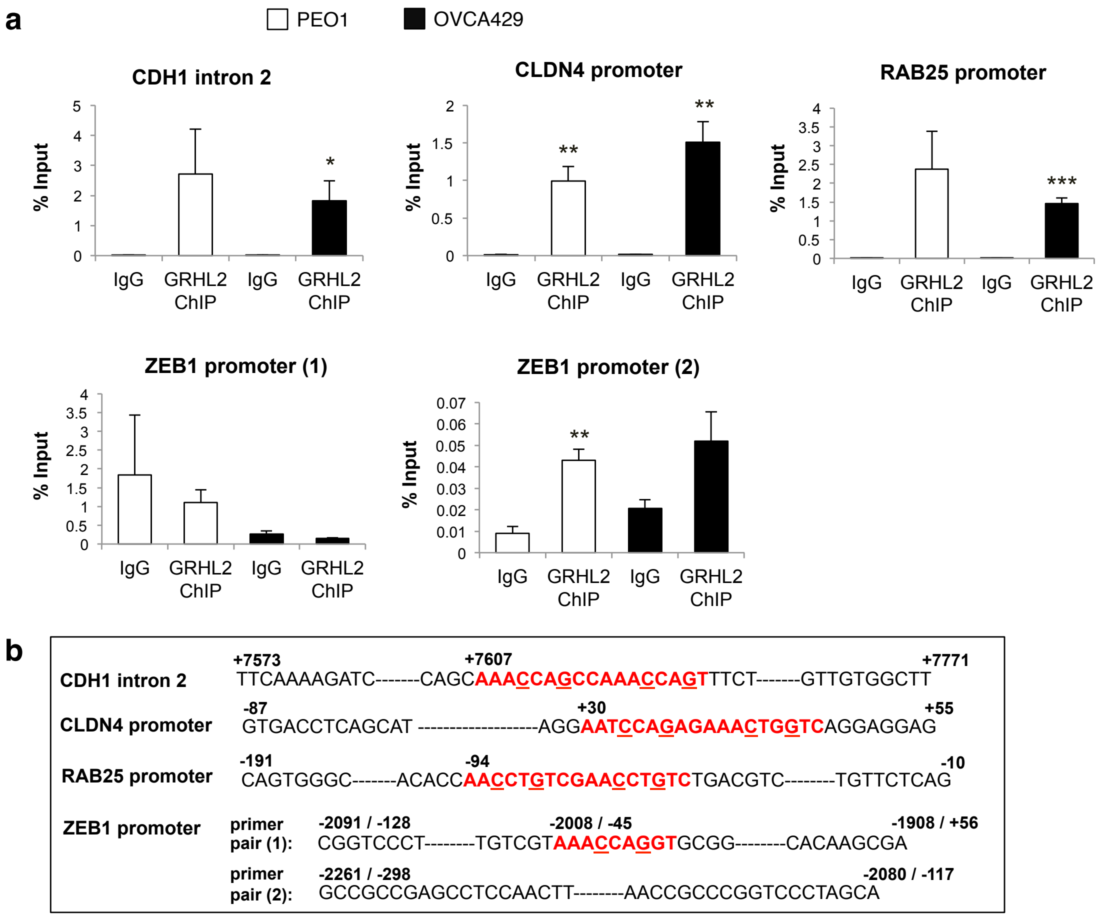


**Supplementary Figure S6. The binding of GRHL2 at the regulatory elements of *CDH1, CLDN4, RAB25* and *ZEB1* genes.**

(**a**) GRHL2 ChIP-qPCR in PEO1 and OVCA429 at intron 2 of *CDH1* and the promoter regions of *CLDN4*, *RAB25* and *ZEB1*. Signals of IgG control and ChIP samples were normalized to input DNA and presented as % input with SEM from three independent experiments. % input of GRHL2 ChIP samples were compared with IgG control using unpaired *t*-tests. * $p < 0.05$; ** $p < 0.01$; *** $p < 0.001$. Two primer pairs were tested for ZEB1 promoter and no enrichment was detected by primer pair 1. (**b**) DNA sequences (enhancer/promoter) of different genes flanked by the primer pairs used in ChIP-qPCR. The GRHL2 binding motifs are labelled in red. Numbers refer to the distance (in base pairs) relative to the transcription start site (TSS) of each gene. For *ZEB1*, the distance relative to the TSS of both NM_001128128 and NM_030751 gene transcripts are indicated.

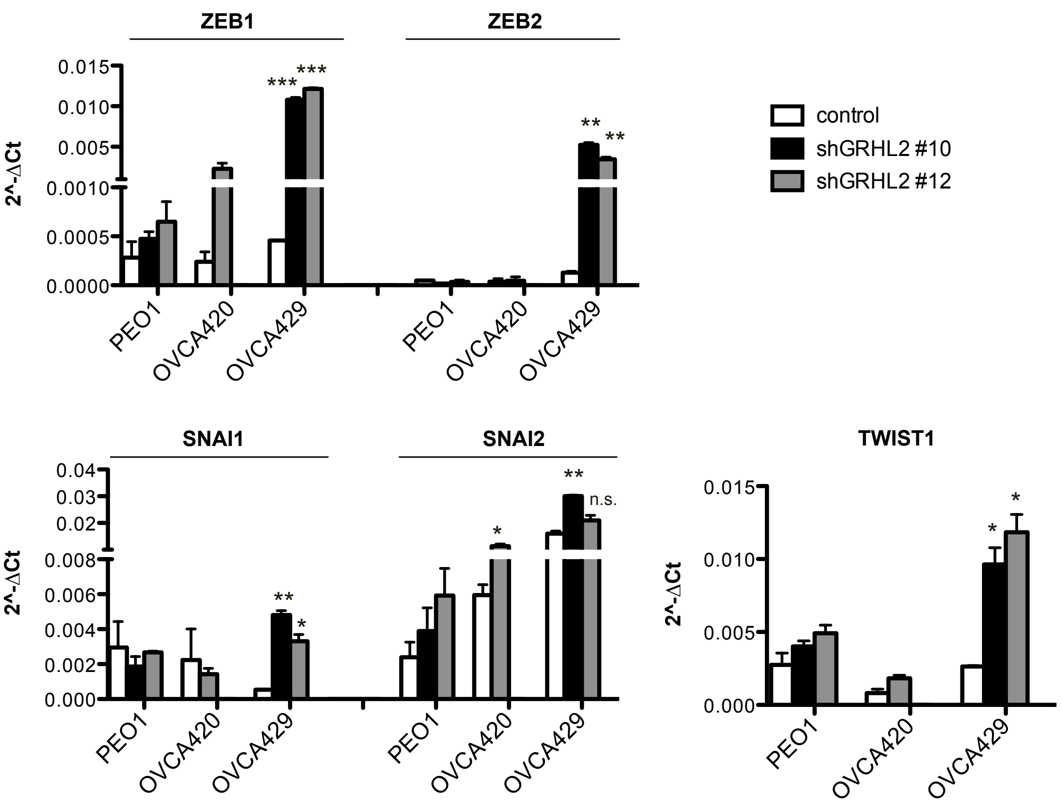


**Supplementary Figure S7. Knockdown of GRHL2 affects the mRNA expression of other EMT transcription factors.**

Bar charts showing the normalized mRNA expression ($2^{-\Delta Ct}$) (*y*-axis) of five EMT transcriptional drivers (ZEB1, ZEB2, SNAI1, SNAI2 and TWIST1) measured by RT-qPCR in control (white bars), shGRHL2 #10 (black bars), and shGRHL2 #12 (grey bars) cells of PEO1, OVCA420 and OVCA429. Unpaired *t*-tests were performed for statistical significance. * $p < 0.05$; ** $p < 0.01$; *** $p < 0.001$.

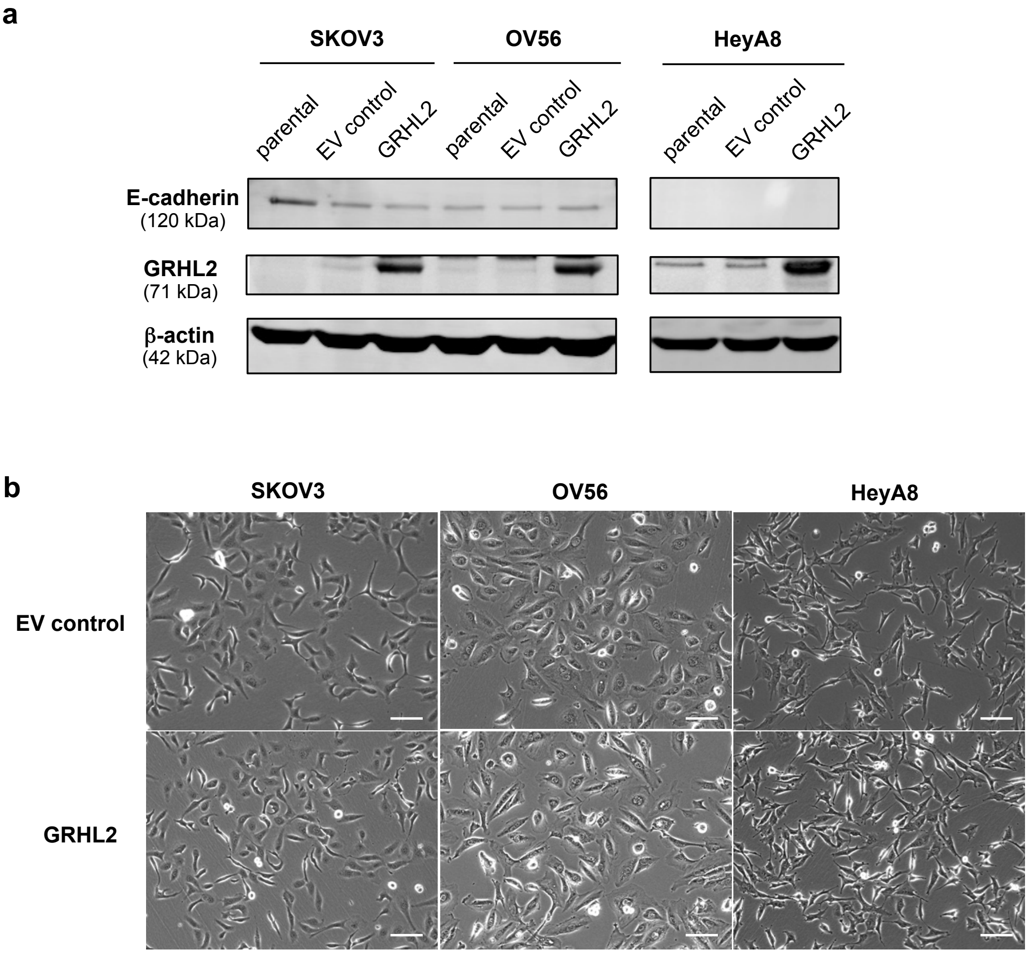


**Supplementary Figure S8. Ectopic overexpression of GRHL2 in SKOV3, OV56 and HeyA8 cell lines.**

(**a**) Western blots of E-cadherin, GRHL2 and β-actin in parental, empty vector (EV) control, and GRHL2-overexpressing cells of three cell lines: SKOV3, OV56, HeyA8. Full-length blots are presented in Supplementary Figure S10. (**b**) Phase-contrast images showing the cell morphologies of SKOV3, OV56, HeyA8 cells infected with empty vector (EV) control and GRHL2-overexpressing plasmids. Scale = 100 μm.

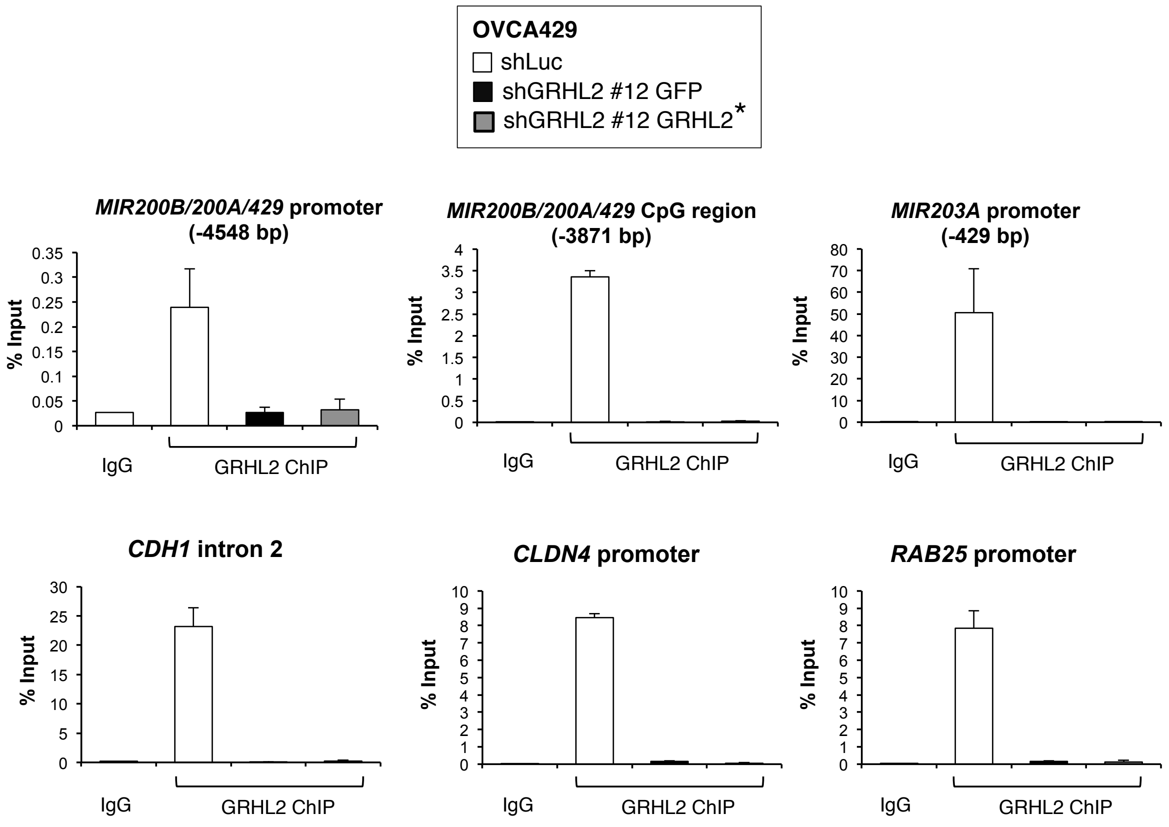


**Supplementary Figure S9. The binding of GRHL2 at its target sites in OVCA429 control, shGRHL2 #12 GFP and GRHL2*-overexpressed shGRHL2 #12 cells.** GRHL2 ChIP-qPCR in OVCA429 shLuc, shGRHL2 #12 GFP and shGRHL2 #12 GRHL2* cells at the regulatory elements of *MIR200B/200A/429*, *MIR203A*, *CDH1*, *CLDN4*, and *RAB25*. Signals of IgG control and ChIP samples were normalized to input DNA and presented as % input.

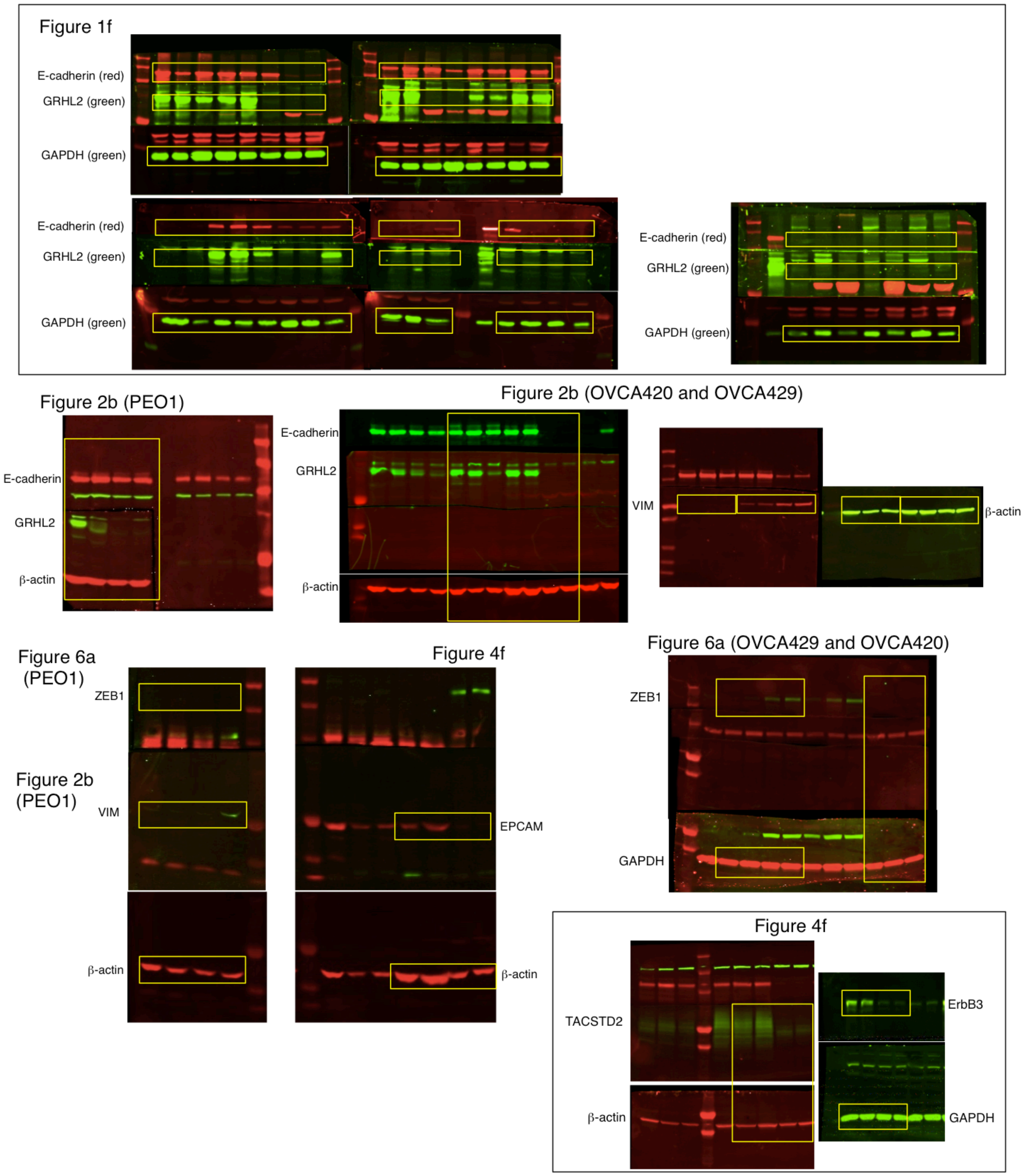


**Supplementary Figure S10. Full-length blots.**

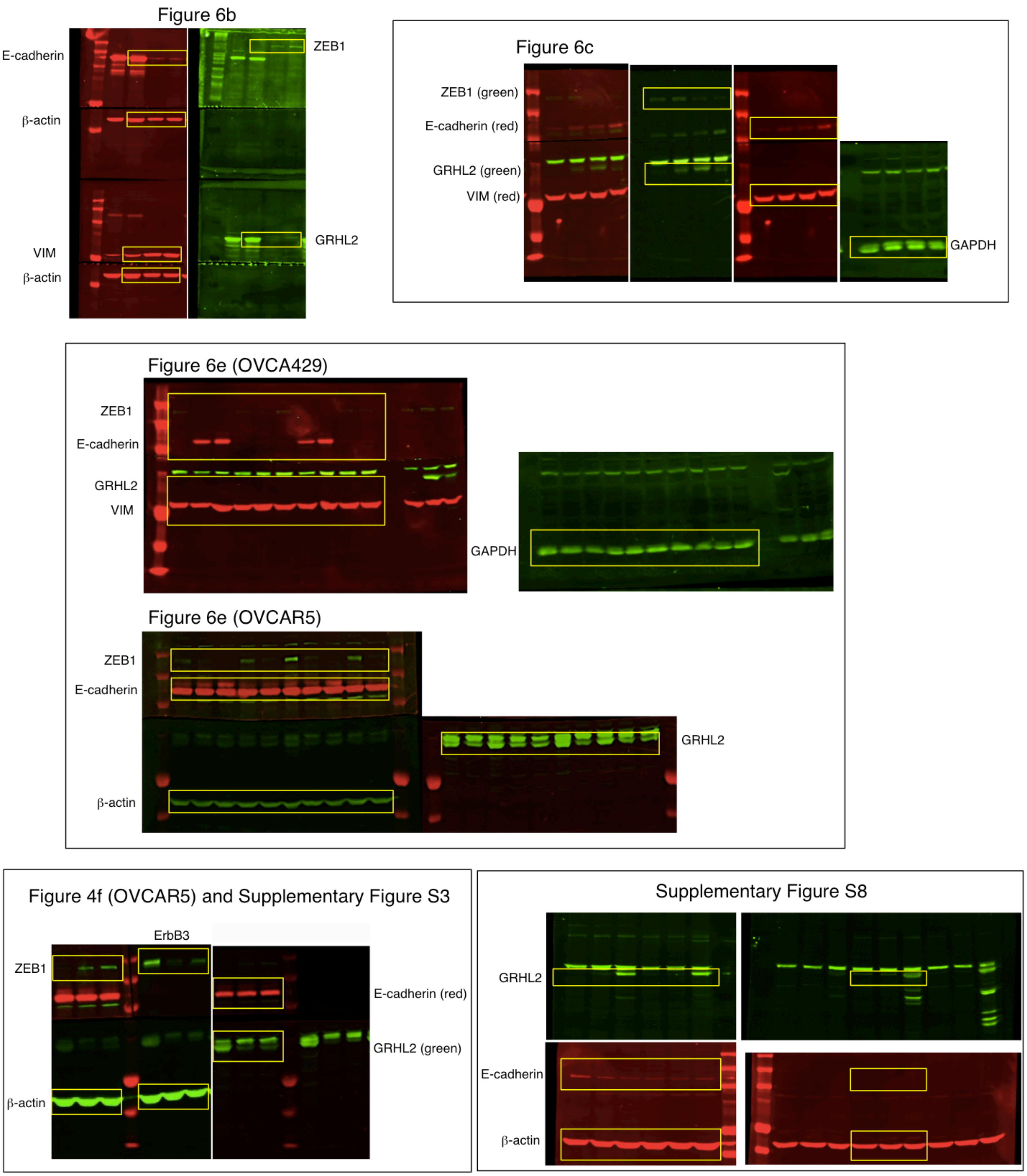


**Supplementary Figure S10 (continued).**

## Supplementary Tables

**Supplementary Table S1. Expression changes of molecular subtype and EMT signature genes in GRHL2-knockdown OVCA429.**

Table shows the mean log2 fold change of molecular subtype and EMT signature genes in shGRHL2 #10 and shGRHL2 #12 (5 samples in total) OVCA429 cells compared to control (shNon) based on GeneChip® Human Gene 2.0 ST Array.

| probeset | Gene | Mean log2 fold change | Consistency in 5 samples | Subtype signatures | EMT signatures |
|---|---|---|---|---|---|
| 16703715 | *ZEB1* | 0.379867472 | 5 | Mes_Up | Mes |
| 17049676 | *SERPINE1* | 1.360756325 | 5 | Mes_Up | --- |
| 16897719 | *CCDC88A* | 1.368775499 | 5 | EpiA_Dn | Mes |
| 17078610 | *IMPA1* | 0.538482868 | 5 | EpiA_Dn | Mes |
| 16755878 | *CHPT1* | 0.43543463 | 5 | EpiA_Dn | --- |
| 16832147 | *ALDH3A2* | 0.312655046 | 5 | EpiA_Dn | --- |
| 17014839 | *C6orf120* | 0.640869627 | 5 | --- | Mes |
| 17005138 | *CAP2* | 0.297158459 | 5 | --- | Mes |
| 16854437 | *CDH2* | 0.793885546 | 5 | --- | Mes |
| 16800871 | *EID1* | 0.614386046 | 5 | --- | Mes |
| 16970404 | *FGF2* | 1.191353807 | 5 | --- | Mes |
| 16906835 | *PGAP1* | 0.660551645 | 5 | --- | Mes |
| 16691046 | *PHTF1* | 0.406282278 | 5 | --- | Mes |
| 16792226 | *SEC23A* | 0.371906483 | 5 | --- | Mes |
| 17113980 | *SMARCA1* | 0.836271972 | 5 | --- | Mes |
| 16790267 | *SUPT16H* | 0.463567083 | 5 | --- | Mes |
| 16702767 | *VIM* | 0.73864527 | 5 | --- | Mes |
| 16968331 | *FGF5* | 0.389371311 | 4 | Mes_Up | --- |
| 16896346 | *FEZ2* | 0.220701263 | 4 | Mes_Up | --- |
| 17050591 | *MET* | 0.568297792 | 4 | EpiA_Up | --- |
| 17056426 | *PDE1C* | 0.415867346 | 4 | EpiA_Up | --- |
| 16878414 | *MRPL33* | 0.235583493 | 4 | EpiA_Up | --- |
| 16780808 | *KDELC1* | 0.26957135 | 4 | EpiA_Dn | Mes |
| 16850477 | *TYMS* | 1.173964775 | 4 | EpiA_Dn | --- |
| 16828450 | *CFDP1* | 0.474727562 | 4 | EpiA_Dn | --- |
| 16812717 | *BTBD1* | 0.292358516 | 4 | EpiA_Dn | --- |
| 16943681 | *DZIP3* | 0.268736522 | 4 | EpiA_Dn | --- |
| 17117484 | *TACC2* | 0.246212394 | 4 | EpiA_Dn | --- |
| 16905292 | *CHN1* | 0.310677891 | 4 | --- | Mes |
| 16966649 | *DCUN1D4* | 0.487320578 | 4 | --- | Mes |
| 16847321 | *HEATR6* | 0.341561859 | 4 | --- | Mes |
| 16981364 | *NEK1* | 0.271764004 | 4 | --- | Mes |
| 16806914 | *NOP10* | 0.40776221 | 4 | --- | Mes |
| 16772493 | *STX2* | 0.550816438 | 4 | --- | Mes |
| 16675334 | *TROVE2* | 0.583041148 | 4 | --- | Mes |
| 16946513 | *TRPC1* | 0.43825924 | 4 | --- | Mes |
| 16919567 | *SDC4* | 0.393103945 | 4 | --- | Epi |
| 16754373 | *GLIPR1* | 0.271395018 | 3 | Mes_Up | --- |
| 16840318 | *NLRP1* | 0.231051027 | 3 | Mes_Up | --- |
| 16731461 | *NNMT* | 0.166710594 | 3 | Mes_Up | --- |
| 16950989 | *WNT7A* | 0.213921448 | 3 | EpiA_Up | --- |
| 16765389 | *ATP5G2* | 0.258602723 | 3 | EpiA_Dn | Mes |
| 16708179 | *CUTC* | 0.254735074 | 3 | EpiA_Dn | Mes |
| 16812011 | *HMG20A* | 0.190601096 | 3 | EpiA_Dn | Mes |
| 16955511 | *PDHB* | 0.21342514 | 3 | EpiA_Dn | Mes |
| 17022529 | *WASF1* | 0.329399999 | 3 | EpiA_Dn | Mes |
| 16781695 | *ARHGEF40* | 0.227221572 | 3 | --- | Mes |
| 16840902 | *AURKB* | 0.293902814 | 3 | --- | Mes |
| 16868130 | *CD320* | 0.157148038 | 3 | --- | Mes |
| 16939247 | *EXOG* | 0.144691024 | 3 | --- | Mes |
| 16690139 | *EXTL2* | 0.239413911 | 3 | --- | Mes |

| | | | | | |
|---|---|---|---|---|---|
| 17087191 | *HABP4* | 0.451069501 | 3 | --- | Mes |
| 16701324 | *HNRNPU* | 0.300494888 | 3 | --- | Mes |
| 16703068 | *MLLT10* | 0.216686901 | 3 | --- | Mes |
| 17022333 | *OSTM1* | 0.350622677 | 3 | --- | Mes |
| 16970673 | *PHF17* | 0.135257405 | 3 | --- | Mes |
| 16855026 | *PIAS2* | 0.465384287 | 3 | --- | Mes |
| 16987125 | *POLR3G* | 0.302812326 | 3 | --- | Mes |
| 17014562 | *QKI* | 0.188964432 | 3 | --- | Mes |
| 16721861 | *SWAP70* | 0.487974308 | 3 | --- | Mes |
| 16866724 | *TCF3* | 0.253140361 | 3 | --- | Mes |
| 16869876 | *WIZ* | 0.089881887 | 3 | --- | Mes |
| 17097375 | *ZFP37* | 0.217851497 | 3 | --- | Mes |
| 16663051 | *ZNF643* | 0.246162986 | 3 | --- | Mes |
| 16961616 | *TNFSF10* | 0.302585532 | 3 | --- | Epi |
| 16846157 | *PRR15L* | 0.263856245 | 3 | --- | Epi |
| 16841662 | *TRIM16* | 0.099878654 | 3 | --- | Epi |
| 17084669 | *RUSC2* | -0.174938576 | -3 | Mes_Up | --- |
| 16865590 | *EPS8L1* | -0.123828372 | -3 | EpiA_Up | Epi |
| 16799690 | *SPINT1* | -0.153667178 | -3 | EpiA_Up | Epi |
| 16733473 | *ST14* | -0.160362514 | -3 | EpiA_Up | Epi |
| 16991991 | *WWC1* | -0.184467935 | -3 | EpiA_Up | Epi |
| 16727232 | *CST6* | -0.250536375 | -3 | EpiA_Up | Epi |
| 17079477 | *STK3* | -0.15998884 | -3 | EpiA_Up | --- |
| 16666336 | *ST6GALNAC5* | -0.16982067 | -3 | EpiA_Up | --- |
| 16918755 | *FER1L4* | -0.187311181 | -3 | EpiA_Up | --- |
| 16919399 | *JPH2* | -0.220328153 | -3 | EpiA_Up | --- |
| 16702351 | *GATA3* | -0.23649814 | -3 | EpiA_Up | --- |
| 16823719 | *FAM86A* | -0.150107505 | -3 | EpiA_Dn | --- |
| 16705051 | *CISD1* | -0.185121804 | -3 | EpiA_Dn | --- |
| 16719088 | *NSMCE4A* | -0.235934966 | -3 | EpiA_Dn | --- |
| 16840284 | *C1QBP* | -0.213699476 | -3 | --- | Mes |
| 16845219 | *CCR10* | -0.257962531 | -3 | --- | Mes |
| 16924719 | *CCT8* | -0.169012479 | -3 | --- | Mes |
| 16712991 | *MTPAP* | -0.181114525 | -3 | --- | Mes |
| 16672779 | *PPOX* | -0.163075447 | -3 | --- | Mes |
| 17015277 | *RPP40* | -0.22916957 | -3 | --- | Mes |
| 16977016 | *SDAD1* | -0.320691444 | -3 | --- | Mes |
| 17009193 | *SLC29A1* | -0.201386037 | -3 | --- | Mes |
| 16867088 | *TBXA2R* | -0.189541146 | -3 | --- | Mes |
| 16823666 | *PPL* | -0.144019778 | -3 | --- | Epi |
| 16898788 | *TGFA* | -0.147428052 | -3 | --- | Epi |
| 16753030 | *PIP4K2C* | -0.172588124 | -3 | --- | Epi |
| 16720268 | *EPS8L2* | -0.177340409 | -3 | --- | Epi |
| 17030426 | *DDR1* | -0.193237412 | -3 | --- | Epi |
| 17033131 | *DDR1* | -0.193949128 | -3 | --- | Epi |
| 17037907 | *DDR1* | -0.193949128 | -3 | --- | Epi |
| 17027581 | *DDR1* | -0.21066188 | -3 | --- | Epi |
| 16861630 | *SPINT2* | -0.210694707 | -3 | --- | Epi |
| 16695490 | *F11R* | -0.265460921 | -3 | --- | Epi |
| 16745002 | *MPZL2* | -0.287830396 | -3 | --- | Epi |
| 17050522 | *TES* | -0.319898883 | -3 | --- | Epi |
| 16662322 | *GJB3* | -0.215511195 | -4 | EpiA_Up | Epi |
| 16664218 | *TSPAN1* | -0.231627778 | -4 | EpiA_Up | Epi |
| 16893709 | *SH3YL1* | -0.261561085 | -4 | EpiA_Up | Epi |
| 16840599 | *CLDN7* | -0.304040841 | -4 | EpiA_Up | Epi |
| 17010461 | *MYO6* | -0.777588228 | -4 | EpiA_Up | Epi |
| 16869643 | *GIPC1* | -0.294236919 | -4 | EpiA_Up | --- |
| 16764817 | *KRT81* | -0.479493411 | -4 | EpiA_Up | --- |
| 16692724 | *ANP32E* | -0.225426717 | -4 | --- | Mes |
| 16660503 | *CDC42* | -0.24501568 | -4 | --- | Mes |
| 16778688 | *NUFIP1* | -0.286294865 | -4 | --- | Mes |
| 16769007 | *UHRF1BP1L* | -0.263837071 | -4 | --- | Mes |
| 16833876 | *GRB7* | -0.179310548 | -4 | --- | Epi |
| 16908338 | *TMBIM1* | -0.187492717 | -4 | --- | Epi |

| | | | | | |
|---|---|---|---|---|---|
| 16661314 | *SFN* | -0.201540248 | -4 | --- | Epi |
| 16733288 | *ST3GAL4* | -0.210965169 | -4 | --- | Epi |
| 17024187 | *PERP* | -0.247570088 | -4 | --- | Epi |
| 16674130 | *KIAA0040* | -0.257499416 | -4 | --- | Epi |
| 16844775 | *KRT19* | -0.307649391 | -4 | --- | Epi |
| 16968213 | *ANXA3* | -0.554525896 | -4 | --- | Epi |
| 16860946 | *LSR* | -0.351938743 | -5 | Mes_Dn | Epi |
| 17058601 | *CLDN3* | -0.260280288 | -5 | EpiA_Up | Epi |
| 17071497 | *GRHL2* | -0.281223533 | -5 | EpiA_Up | Epi |
| 16874693 | *KLK6* | -0.288243544 | -5 | EpiA_Up | Epi |
| 17107309 | *VGLL1* | -0.380250773 | -5 | EpiA_Up | Epi |
| 16904551 | *GALNT3* | -0.444640436 | -5 | EpiA_Up | Epi |
| 16665373 | *INADL* | -0.490899515 | -5 | EpiA_Up | Epi |
| 16957304 | *ZBED2* | -0.519165498 | -5 | EpiA_Up | Epi |
| 17070949 | *ESRP1* | -0.523708165 | -5 | EpiA_Up | Epi |
| 16820486 | *CDH1* | -0.579192164 | -5 | EpiA_Up | Epi |
| 17089525 | *LCN2* | -0.817789812 | -5 | EpiA_Up | Epi |
| 16904193 | *ITGB6* | -1.874703719 | -5 | EpiA_Up | Epi |
| 16778849 | *LCP1* | -0.706980921 | -5 | EpiA_Up | --- |
| 17043982 | *ITGB8* | -1.043347754 | -5 | EpiA_Up | --- |
| 16984689 | *ITGA2* | -1.068027801 | -5 | EpiA_Up | --- |
| 16858321 | *CARM1* | -0.233360651 | -5 | --- | Mes |
| 16820737 | *DDX19A* | -0.514021193 | -5 | --- | Mes |
| 16671901 | *RAB25* | -0.242807794 | -5 | --- | Epi |
| 16965915 | *C4orf19* | -0.2486128 | -5 | --- | Epi |
| 16748529 | *GPRC5A* | -0.299486836 | -5 | --- | Epi |
| 17024053 | *MAP7* | -0.333257656 | -5 | --- | Epi |
| 16820463 | *CDH3* | -0.368637439 | -5 | --- | Epi |
| 16723569 | *EHF* | -0.382435719 | -5 | --- | Epi |
| 16838330 | *SYNGR2* | -0.471763344 | -5 | --- | Epi |
| 16775546 | *SCEL* | -0.873303915 | -5 | --- | Epi |
| 16689869 | *F3* | -0.895222509 | -5 | --- | Epi |
| 16687847 | *TACSTD2* | -1.012206028 | -5 | --- | Epi |
| 17011302 | *AIM1* | -1.401691436 | -5 | --- | Epi |
| 17117110 | *CD24* | -1.819563843 | -5 | --- | Epi |
| 16879863 | *EPCAM* | -2.117373855 | -5 | --- | Epi |

**Supplementary Table S2. GRHL2 target genes with their respective GRHL2 binding sites identified by ChIP-seq in EOC cells.**

Table shows the GRHL2 ChIP-seq peaks associated with *ARHGEF19, CDH1, CLDN4, DRAM1, EEA1, ELF3, EPCAM, EPS8, ERBB3, ESRP1/2, GRHL1/2, MAL2, PRSS8, SLC44A2, SPINT1, RAB25, ST14, TACSTD2* and *ZEB1* genes detected in OVCAR3, PEO1 and OVCA429, with their respective peak length, MACS (Model-based analysis of ChIP-seq) score, fold enrichment, distance to TSS and genomic annotation. ChIP-seq peaks validated by ChIP-qPCR (Supplementary Figure S5) are marked ■.

| Gene | Cell line | No. of Peaks | Peak Length | Score | Enrichment | Distance to TSS (base) | Genomic Annotation | |
|---|---|---|---|---|---|---|---|---|
| ***ARHGEF19*** | OVCAR3 | 1 | 292 | 71 | 54.62 | -138 | Promoter | |
| | PEO1 | 1 | 293 | 41 | 75.26 | -137 | Promoter | |
| | OVCA429 | 1 | 486 | 136 | 65.95 | -147 | Promoter | |
| ***CDH1*** | OVCAR3 | 1 | 282 | 25 | 68.02 | 7602 | Intron 2 | ■ |
| | PEO1 | 1 | 594 | 126 | 231.28 | 7621 | Intron 2 | ■ |
| | OVCA429 | 3 | 749 | 179 | 260.42 | 7624 | Intron 2 | ■ |
| | | | 616 | 35 | 51.96 | 41716 | Intron 2 | |
| | | | 253 | 32 | 39.12 | 52942 | Intron 2 | |
| ***CLDN4*** | OVCAR3 | 2 | 205 | 33 | 50.77 | -5672 | Intergenic | |
| | | | 328 | 37 | 56.93 | 1525 | 3’ UTR | |
| | PEO1 | 4 | 326 | 73 | 58.37 | -5668 | Intergenic | |
| | | | 658 | 62 | 51.32 | 45 | 5’ UTR | ■ |
| | | | 578 | 203 | 162.32 | 1514 | 3’ UTR | |
| | | | 299 | 34 | 34.06 | 2659 | TTS | |
| | OVCA429 | 5 | 395 | 158 | 173.75 | -5680 | Intergenic | |
| | | | 317 | 73 | 65.97 | -1140 | Intergenic | |
| | | | 678 | 126 | 87.29 | 20 | 5’ UTR | ■ |
| | | | 586 | 174 | 84.38 | 1509 | 3’ UTR | |
| | | | 488 | 25 | 25.85 | 2639 | TTS | |
| ***DRAM1*** | PEO1 | 2 | 280 | 38 | 24.93 | 194 | 5’ UTR | |
| | | | 482 | 72 | 112.75 | 17581 | Intron 1 | |
| | OVCA429 | 3 | 392 | 64 | 55.42 | 191 | 5’ UTR | |
| | | | 398 | 69 | 50.19 | 17597 | Intron 1 | |
| | | | 193 | 26 | 37.83 | 31251 | Intron 4 | |
| ***EEA1*** | OVCAR3 | 1 | 233 | 27 | 41.54 | -488 | Promoter | |
| | PEO1 | 1 | 505 | 109 | 87.16 | -493 | Promoter | |
| | OVCA429 | 1 | 608 | 149 | 54.19 | -474 | Promoter | |
| ***ELF3*** | OVCAR3 | 4 | 241 | 67 | 103.09 | -2240 | Intergenic | |
| | | | 490 | 45 | 69.24 | -158 | Promoter | |
| | | | 355 | 92 | 70.78 | 24416 | Intergenic | |
| | | | 377 | 132 | 187.1 | 35892 | Intergenic | |
| | PEO1 | 4 | 607 | 191 | 114.54 | -2235 | Intergenic | |
| | | | 705 | 111 | 80.39 | -155 | Promoter | |
| | | | 342 | 195 | 154.07 | 24414 | Intergenic | |
| | | | 493 | 220 | 75.39 | 35895 | Intergenic | |
| | OVCA429 | 6 | 541 | 205 | 99.42 | -2239 | Intergenic | |
| | | | 499 | 118 | 116.79 | -149 | Promoter | |
| | | | 453 | 149 | 54.19 | 24416 | Intergenic | |
| | | | 543 | 241 | 175.31 | 35890 | Intergenic | |
| | | | 455 | 29 | 21.1 | 46803 | Intergenic | |

| | | | | | | | |
|---|---|---|---|---|---|---|---|
| | | | 252 | 40 | 51.96 | 90650 | Intergenic |
| ***EPCAM*** | PEO1 | 1 | 675 | 87 | 100.82 | -29143 | Intergenic |
| | OVCA429 | 1 | 664 | 82 | 114.38 | -29133 | Intergenic |
| ***EPS8*** | PEO1 | 1 | 574 | 108 | 43.18 | -136 | Promoter |
| | OVCA429 | 1 | 784 | 114 | 82.93 | -145 | Promoter |
| ***ERBB3*** | OVCAR3 | 1 | 617 | 31 | 60.64 | -213 | Promoter |
| | PEO1 | 1 | 1167 | 149 | 132.82 | -248 | Promoter |
| | OVCA429 | 2 | 1224 | 197 | 157.48 | -247 | Promoter |
| | | | 334 | 59 | 72.99 | 6197 | TTS (variant s) |
| ***ESRP1*** | OVCA429 | 1 | 290 | 52 | 25.22 | 16087 | Intron 4 |
| ***ESRP2*** | OVCAR3 | 1 | 585 | 119 | 176.05 | 2019 | Intron 3 |
| | PEO1 | 1 | 575 | 101 | 121.14 | 2031 | Intron 3 |
| | OVCA429 | 1 | 819 | 158 | 114.94 | 2029 | Intron 3 |
| ***GRHL1*** | OVCAR3 | 1 | 284 | 67 | 34.36 | 147 | 5'UTR |
| | PEO1 | 3 | 358 | 177 | 84.92 | -9874 | Intergenic |
| | | | 327 | 78 | 31.18 | 141 | 5'UTR |
| | | | 250 | 72 | 59.6 | 40585 | Intron 11 |
| | OVCA429 | 4 | 352 | 42 | 48.5 | -9864 | Intergenic |
| | | | 322 | 80 | 29.1 | 80 | 5'UTR |
| | | | 316 | 43 | 59.19 | 19600 | Intron 8 |
| | | | 335 | 61 | 72.86 | 40581 | Intron 11 |
| ***GRHL2*** | OVCAR3 | 3 | 361 | 119 | 202.65 | -55188 | Intergenic |
| | | | 339 | 68 | 108.76 | -53813 | Intergenic |
| | | | 468 | 133 | 204.63 | 182 | 5'UTR |
| | PEO1 | 4 | 667 | 186 | 111.54 | -55176 | Intergenic |
| | | | 264 | 25 | 14.49 | -53809 | Intergenic |
| | | | 1001 | 271 | 108.35 | 228 | 5'UTR |
| | | | 279 | 30 | 17.99 | 12293 | Intron 1 |
| | OVCA429 | 3 | 637 | 135 | 175.36 | -55149 | Intergenic |
| | | | 411 | 33 | 36.1 | -53863 | Intergenic |
| | | | 763 | 248 | 257.72 | 250 | 5'UTR |
| ***MAL2*** | PEO1 | 4 | 458 | 31 | 29.13 | -64135 | Intergenic |
| | | | 428 | 73 | 35.02 | -7461 | Intergenic |
| | | | 672 | 145 | 69.56 | -3015 | Intergenic |
| | | | 254 | 40 | 23.99 | -1070 | Intergenic |
| | OVCA429 | 1 | 403 | 66 | 76.21 | -3024 | Intergenic |
| ***PRSS8*** | OVCAR3 | 2 | 281 | 59 | 171.3 | 4795 | TTS of *PRSS8* |
| | | | 439 | 35 | 53.85 | -3238 | TTS of *PRSS36* |
| | PEO1 | 2 | 837 | 216 | 64.77 | 4819 | TTS of *PRSS8* |
| | | | 534 | 113 | 105.04 | -3238 | TTS of *PRSS36* |
| | OVCA429 | 2 | 958 | 184 | 239.01 | 4816 | TTS of *PRSS8* |
| | | | 800 | 176 | 250.55 | -3243 | TTS of *PRSS36* |
| ***SLC44A2*** | OVCAR3 | 1 | 345 | 183 | 270.73 | -4 | Promoter |
| | PEO1 | 2 | 528 | 147 | 88.16 | -7599 | Intron 1 of isoform 2 |
| | | | 990 | 275 | 131.93 | -9 | Promoter |
| | OVCA429 | 3 | 595 | 64 | 95.01 | -21967 | Intron 1 of isoform 2 |
| | | | 521 | 127 | 184.77 | -7592 | Intron 1 of isoform 2 |
| | | | 780 | 278 | 67.41 | -4 | Promoter |
| ***SPINT1*** | OVCAR3 | 3 | 478 | 72 | 110.78 | 13297 | 3'UTR of *SPINT1* |

| | | | | | | | | |
|---|---|---|---|---|---|---|---|---|
| | | | 280 | 35 | 26.93 | 27523 | Intergenic | |
| | | | 290 | 54 | 70.23 | 27956 | 3'UTR of *RHOV* | |
| | PEO1 | 4 | 620 | 180 | 136.63 | 13296 | 3'UTR of *SPINT1* | |
| | | | 389 | 31 | 18.59 | 21614 | Intergenic | |
| | | | 137 | 31 | 18.59 | 27524 | Intergenic | |
| | | | 281 | 40 | 23.99 | 27961 | 3'UTR of *RHOV* | |
| | OVCA429 | 2 | 611 | 179 | 130.21 | 13308 | 3'UTR of *SPINT1* | |
| | | | 803 | 67 | 89.84 | 27956 | 3'UTR of *RHOV* | |
| ***RAB25*** | OVCAR3 | 1 | 379 | 113 | 86.93 | -82 | Promoter | ■ |
| | PEO1 | 1 | 466 | 174 | 163.49 | -92 | Promoter | ■ |
| | OVCA429 | 1 | 520 | 205 | 149.12 | -87 | Promoter | ■ |
| ***ST14*** | OVCAR3 | 1 | 269 | 39 | 60.01 | 412 | Intron 1 | |
| | PEO1 | 3 | 360 | 42 | 50.35 | -13185 | Intergenic | |
| | | | 421 | 73 | 57.68 | 391 | Intron 1 | |
| | | | 311 | 27 | 21.59 | 6790 | Intron 1 | |
| | OVCA429 | 3 | 466 | 46 | 45.53 | -13216 | Intergenic | |
| | | | 359 | 67 | 32.49 | 413 | Intron 1 | |
| | | | 273 | 26 | 30.7 | 6793 | Intron 1 | |
| ***TACSTD2*** | OVCAR3 | 1 | 328 | 91 | 70.01 | 209 | 5' UTR | |
| | PEO1 | 3 | 226 | 40 | 46.35 | 4607 | Intergenic | |
| | | | 611 | 240 | 143.86 | 192 | 5' UTR | |
| | | | 230 | 28 | 13.43 | -15203 | Intergenic | |
| | OVCA429 | 3 | 292 | 53 | 44.06 | 4614 | Intergenic | |
| | | | 796 | 272 | 176.66 | 195 | 5' UTR | |
| | | | 362 | 38 | 37.61 | -42930 | Intergenic | |
| ***ZEB1*** | OVCA429 | 1 | 286 | 25 | 18.19 | 32704 | Intron 1 | |

TSS=Transcription start site; TTS=Transcription termination site; UTR=Untranslated region

**Supplementary Table S3. MicroRNA genes with their respective GRHL2 binding sites identified by ChIP-seq in EOC cells.**

Table shows the GRHL2 ChIP-seq peaks in the vicinity of *MIR203A*, *MIR200B*, *MIR205*, *MIR205HG*, *MIR21*, *MIRLET7G*, *MIR1908*, *MIR4284* genes in OVCAR3, PEO1 and OVCA429 cells with their respective peak length, MACS (Model-based analysis of ChIP-seq) score, fold enrichment, distance to miRNA gene and genomic annotation. ChIP-seq peaks validated by ChIP-qPCR (Figure 5) are marked ■.

| Gene | Cell line | No. of Peaks | Peak Length | Score | Enrichment | Distance to miRNA hairpin (base) | Genomic Annotation | |
|---|---|---|---|---|---|---|---|---|
| ***MIR203A*** | OVCAR3 | 2 | 253 | 54 | 41.54 | -993 | Promoter | |
| | | | 551 | 194 | 208.73 | -369 | Promoter | ■ |
| | PEO1 | 1 | 1090 | 223 | 133.73 | -375 | Promoter | ■ |
| | OVCA429 | 1 | 1189 | 205 | 163.87 | -372 | Promoter | ■ |
| ***MIR200B*** | OVCAR3 | 2 | 325 | 45 | 11.54 | -3846 | CpG | ■ |
| | | | 202 | 25 | 12.82 | -2296 | CpG | |
| | PEO1 | 1 | 458 | 51 | 61.17 | -3859 | CpG | ■ |
| | OVCA429 | 1 | 653 | 41 | 29.82 | -3863 | CpG | ■ |
| ***MIR205*** | PEO1 | 1 | 258 | 33 | 60.57 | 1259 | TTS of MIR205HG | |
| | OVCA429 | 1 | 291 | 59 | 21.46 | 1273 | | |
| ***MIR205HG*** | OVCA429 | 3 | 343 | 65 | 89.47 | -68539 | Intergenic | |
| | | | 294 | 41 | 38.73 | -18767 | Intergenic | |
| | | | 446 | 38 | 44.62 | -12752 | Intergenic | |
| ***MIR205HG/ MIR205*** | OVCA429 | 1 | 399 | 37 | 38.45 | 23 (MIR205HG)<br>-3287 (MIR205) | Promoter of *MIR205HG* | |
| ***MIR21*** | OVCAR3 | 1 | 189 | 33 | 60.09 | -3796 | Intron 10 of gene *VMP1* | |
| | PEO1 | 2 | 801 | 127 | 233.12 | -3798 | | |
| | | | 337 | 38 | 45.58 | 5180 | Intergenic | |
| | OVCA429 | 4 | 521 | 37 | 59.15 | -11695 | Intergenic | |
| | | | 884 | 230 | 334.62 | -3793 | Intron 10 of gene *VMP1* | |
| | | | 349 | 88 | 99.94 | 5167 | Intergenic | |
| | | | 417 | 43 | 20.85 | 16100 | Intergenic | |
| ***MIRLET7G*** | PEO1 | 1 | 243 | 39 | 46.78 | 2821 | Intron 3 of gene *WDR82* | |
| | OVCA429 | 1 | 500 | 148 | 255.93 | 2837 | | |
| ***MIR1908*** | OVCA429 | 1 | 467 | 38 | 40.3 | -117 | Promoter of gene *FADS2* | |
| ***MIR4284*** | OVCA429 | 1 | 293 | 27 | 19.35 | -4685 | Intron 3 of gene *STX1A* | |

TTS=Transcription termination site; HG=Host Gene

**Supplementary Table S4. Differential expression of miR-203, miR-205 and miR-200 family in OVCA429 control and GRHL2-knockdown cells.**

Table shows the log2 fold expression changes (<0 means down-regulation) of miR-200 family members, miR-203 and miR-205, detected from GeneChip® miRNA 2.0 microarray in shGRHL2 #10 and shGRHL2 #12 OVCA429 cells with respect to control (shNon). Only probesets with good detection above background (DABG) signals ($p$ <0.05) are shown. Correlation with GRHL2 expression was checked by Spearman's correlation tests.

| miRNA | Probeset ID | Mean log2 fold change shGRHL2 #10 | Mean log2 fold change shGRHL2 #12 | Mean log2 fold change (combined) | Spearman Rho |
|---|---|---|---|---|---|
| miR-200a-3p | hsa-miR-200a_st | -2.5501 | -4.4951 | -3.5226 | 0.9143 |
| miR-200a-5p | hsa-miR-200a-star_st | -2.9038 | -1.8138 | -2.3588 | 0.3143 |
| miR-200b-3p | hsa-miR-200b_st | -3.3550 | -3.4100 | -3.3825 | 0.8286 |
| precursor of miR-200b | hp_hsa-mir-200b_x_st | -1.2102 | -1.0752 | -1.1427 | 0.7714 |
| miR-429 | hsa-miR-429_st | -0.5309 | -0.5409 | -0.5359 | 0.4571 |
| miR-200c-3p | hsa-miR-200c_st | -2.2310 | -2.7360 | -2.4835 | 0.7333 |
| miR-200c-5p | hsa-miR-200c-star_st | -2.1956 | -2.6356 | -2.4156 | 0.9143 |
| precursor of miR-200c | hp_hsa-mir-200c_st | -1.4083 | -2.0183 | -1.7133 | 0.8286 |
| miR-141 | hsa-miR-141_st | -4.6551 | -5.6601 | -5.1576 | 0.8286 |
| precursor of miR-141 | hp_hsa-mir-141_st | -0.4612 | -0.6362 | -0.5487 | 0.8286 |
| miR-203 | hsa-miR-203_st | -3.0891 | -5.5841 | -4.3366 | 0.8286 |
| miR-205-5p | hsa-miR-205_st | -4.2439 | -2.7839 | -3.5139 | 0.4952 |
| miR-205-3p | hsa-miR-205-star_st | -1.2111 | -1.4111 | -1.3111 | 0.6667 |
| precursor of miR-205 | hp_hsa-mir-205_st | -0.5031 | -0.2231 | -0.3631 | 0.5810 |

**Supplementary Table S5. EOC cell lines with their respective phenotypes and culture media.**

| Cell lines | EMT Phenotype | Media |
|---|---|---|
| A1847 | Mesenchymal | RPMI 1640, 10% FBS, 10 µg/ml insulin |
| A2008 | Epithelial | RPMI 1640, 10% FBS |
| A2780 | Mesenchymal | RPMI 1640, 10% FBS |
| BG1 | Intermediate Mesenchymal | DMEM, 10% FBS, 1 µg/ml insulin |
| C13 | Epithelial | RPMI 1640, 10% FBS |
| Caov2 | Intermediate Epithelial | RPMI 1640, 10% FBS |
| Caov3 | Epithelial | DMEM, 10% FBS |
| CH1 | Intermediate Mesenchymal | DMEM, 10% FBS |
| COLO720E | Mesenchymal | RPMI 1640, 10% FBS |
| DOV13 | Intermediate Mesenchymal | DMEM, 10% FBS |
| EFO21 | Intermediate Epithelial | RPMI 1640, 20% FBS, 1x NEAA |
| FUOV1 | Intermediate Epithelial | DMEM/F12 (1:1), 10% FBS |
| Hey | Intermediate Mesenchymal | RPMI 1640, 10% FBS |
| HeyA8 | Mesenchymal | RPMI 1640, 10% FBS |
| HeyC2 | Intermediate Mesenchymal | RPMI 1640, 10% FBS |
| IGROV1 | Intermediate Epithelial | RPMI 1640, 10% FBS |
| JHOS2 | Intermediate Epithelial | DMEM/F12 (1:1), 10% FBS, 0.1 mM NEAA |
| JHOS3 | Intermediate Epithelial | DMEM/F12 (1:1), 10% FBS, 0.1 mM NEAA |
| JHOS4 | Intermediate Epithelial | DMEM/F12 (1:1), 10% FBS, 0.1 mM NEAA |
| OAW28 | Intermediate Epithelial | DMEM, 10% FBS, 0.7 µg/ml insulin |
| OAW42 | Intermediate Epithelial | DMEM, 10% FBS, 0.7 µg/ml insulin |
| OV17R | Intermediate Epithelial | DMEM/F12 (1:1), 5% FBS, 0.4 µg/ml hyrocortisone, 10 µg/ml insulin |
| OV2008 | Epithelial | RPMI 1640, 10% FBS, 1x NEAA |
| OV56 | Intermediate Epithelial | DMEM/F12 (1:1), 5% FBS, 0.5 µg/ml hyrocortisone, 10 µg/ml insulin |
| OV7 | Intermediate Mesenchymal | DMEM/F12 (1:1), 5% FBS, 0.5 µg/ml hyrocortisone, 10 µg/ml insulin |
| OV90 | Epithelial | MCDB105/M199 (1:1), 10% FBS |
| OVCA420 | Epithelial | DMEM, 10% FBS |
| OVCA429 | Intermediate Epithelial | DMEM, 10% FBS |
| OVCA432 | Intermediate Epithelial | DMEM, 10% FBS |
| OVCA433 | Intermediate Epithelial | DMEM, 10% FBS |
| OVCAR10 | Mesenchymal | RPMI 1640, 10% FBS, 10 µg/ml insulin |
| OVCAR2 | Intermediate Epithelial | RPMI 1640, 10% FBS, 10 µg/ml insulin |
| OVCAR3 | Epithelial | RPMI 1640, 20% FBS, 10 µg/ml insulin |
| OVCAR5 | Intermediate Epithelial | RPMI 1640, 10% FBS, 10 µg/ml insulin |
| OVCAR8 | Epithelial | RPMI 1640, 10% FBS, 10 µg/ml insulin |
| OVK18 | Mesenchymal | DMEM, 10% FBS |
| PEO1 | Epithelial | RPMI 1640, 10% FBS |
| PEO4 | Intermediate Epithelial | RPMI 1640, 10% FBS, 2.5 µg/ml insulin, 1x NEAA |
| SKOV3 | Intermediate Mesenchymal | DMEM, 10% FBS |
| TOV112D | Mesenchymal | MCDB105/M199 (1:1), 10% FBS |
| TykNu | Mesenchymal | DMEM, 10% FBS |
| UWB1.289 | Intermediate Epithelial | RPMI 1640/ MEGM (1:1), 3% FBS |

**Supplementary Table S6. 44 EOC patient samples and their respective molecular subtypes.**

| Tumour Sample | Molecular Subtype |
|---|---|
| JPKO 001 | EpiA |
| JPKO 002 | StemA |
| JPKO 005 | Mes |
| JPKO 007 | StemB |
| JPKO 008 | EpiA |
| JPKO 009 | EpiA |
| JPKO 010 | EpiA |
| JPKO 013 | StemB |
| JPKO 021 | StemB |
| JPKO 023 | StemB |
| JPKO 024 | EpiA |
| JPKO 025 | EpiA |
| JPKO 027 | EpiA |
| JPKO 031 | StemB |
| JPKO 033 | StemB |
| JPKO 036 | StemA |
| JPKO 045 | StemA |
| JPKO 049 | StemA |
| JPKO 055 | EpiA |
| JPKO 056 | EpiA |
| JPKO 060 | StemB |
| JPKO 061 | EpiB |

| Tumour Sample | Molecular Subtype |
|---|---|
| JPKO 063 | StemB |
| JPKO 064 | StemB |
| JPKO 065 | EpiB |
| JPKO 069 | StemA |
| JPKO 070 | EpiA |
| JPKO 079 | Mes |
| JPKO 080 | StemA |
| JPKO 081 | StemA |
| JPKO 082 | EpiB |
| JPKO 083 | Mes |
| JPKO 085 | EpiB |
| JPKO 089 | Mes |
| JPKO 095 | StemB |
| JPKO 098 | Mes |
| JPKO 099 | StemA |
| JPKO 101 | Mes |
| JPKO 102 | Mes |
| JPKO 103 | EpiB |
| JPKO 104 | Mes |
| JPKO 105 | Mes |
| JPKO 106 | EpiB |
| JPKO 110 | EpiB |

**Supplementary Table S7. RT-qPCR primers.**
Primers purchased from Qiagen and used for RT-qPCR in this study.

| Gene | Catalog no. | Ref Seq Accession no. |
|---|---|---|
| *ACTB* | PPH00073E | NM_001101.3 |
| *B2M* | PPH01094E | NM_004048.2 |
| *GAPDH* | PPH00150E | NM_002046.3 |
| *HPRT1* | PPH01018B | NM_000194.2 |
| *RPL13A* | PPH01020B | NM_012423.2 |
| *GRHL1* | PPH19832A | NM_198182.2 |
| *GRHL2* | PPH18929F | NM_024915.3 |
| *GRHL3* | PPH12149A | NM_198174.2 |
| *ZEB1* | PPH01922A | NM_030751.5 |
| *ZEB2* | PPH09021B | NM_014795 |
| *SNAI1* | PPH02459B | NM_005985 |
| *SNAI2* | PPH02475A | NM_003068 |
| *TWIST1* | PPH02132A | NM_000474.3 |
| *CDH1* | PPH00135E | NM_004360.3 |
| *CDH2* | PPH00636F | NM_001792.3 |
| *VIM* | PPH00417E | NM_003380.3 |
| *CLDN4* | PPH07330D | NM_001305.3 |
| *RAB25* | PPH12800E | NM_020387.2 |
| *TACSTD2* | PPH05688A | NM_002353.2 |
| *ERBB3* | PPH00463B | NM_001982.3 |
| *ELF3* | PPH09786C | NM_004433.4 |
| *EPS8* | PPH07120A | NM_004447 |
| *ST14* | PPH09550A | NM_021978.3 |
| *EPCAM* | PPH05720A | NM_002354.2 |
| *MAL2* | PPH10025A | NM_052886.2 |
| *ESRP1* | PPH088556A | NM_017697.3 |
| *ESRP2* | PPH18162A | NM_024939.2 |
| *EEA1* | PPH07165A | NM_003566 |
| *DRAM1* | PPH19768F | NM_018370.2 |
| *ARHGEF19* | PPH22922A | NM_153213.3 |

| Gene | Name of gene product | Catalog no. |
|---|---|---|
| *RNU6-6P* | U6 snRNA 6, pseudogene | MS00033740 |
| *MIR200A* | hsa-miR-200a-3p | MS00003738 |
| *MIR200B* | hsa-miR-200b-3p | MS00009016 |
| | hsa-miR-200b-5p | MS00009023 |
| *MIR200C* | hsa-miR-200c-3p | MS00003752 |
| *MIR203A* | hsa-miR-203a-3p | MS00003766 |
| *MIR203B* | hsa-miR-203b-5p | MS00042217 |
| *MIR205* | hsa-miR-205-5p | MS00003780 |

**Supplementary Table S8. ChIP-qPCR primers.**

| | |
|---|---|
| *CDH1* intron 2 | (forward) 5' TTCAAAAGATCCCCTGCGCT 3' |
| | (reverse) 5' AAGCCACAACAAACCCGTTC 3' |
| *RAB25* promoter | (forward) 5' CAGTGGGCTGTCTCTGAAGG 3' |
| | (reverse) 5' CTGAGAACAGGAAGAGCGGG 3' |
| *CLDN4* promoter | (forward) 5' GTGACCTCAGCATGGGCTTTGA 3’ |
| | (reverse) 5' CTCCTCCTGACCAGTTTCTCTG 3’ |
| *ZEB1* promoter_1 | (forward) 5' CGGTCCCTAGCAACAAGGTT 3’ |
| | (reverse) 5' TCGCTTGTGTCTAAATGCTCG 3' |
| *ZEB1* promoter_2 | (forward) 5' GCCGCCGAGCCTCCAACTTT 3' |
| | (reverse) 5' TGCTAGGGACCGGGCGGTTT 3' |
| *MIR200B/200A/429* promoter (-4548 bp) | |
| | (forward) 5’AGGTGGAGAGGCGAGAGTTGC 3’ |
| | (reverse) 5’ CCAGGATGGGAAGGCTTCTGTG 3’ |
| *MIR200B/200A/429* CpG region (-3871 bp) | |
| | (forward) 5’ CGCAGCAGTGGAACCTGT 3’ |
| | (reverse) 5’ GACAGCCCATCTGTCACCTG 3’ |
| *MIR203A* promoter (-429 bp) | |
| | (forward) 5’ GGCCGTGGAGGATCAGTCG 3’ |
| | (reverse) 5’ CTTCCCGGCGCCGGAATGT 3’ |
